# Individual differences in prosocial learning are represented in the hippocampal dorsal CA1

**DOI:** 10.1038/s41593-026-02292-2

**Published:** 2026-05-12

**Authors:** Filippo La Greca, Elisa Zianni, Ilaria Cerchiaro, Michela Gusmini, Giulia Coccia, Alexandre Carrea, Silvia Pelucchi, Carlo Castoldi, Davide Maggioni, Elena Marcello, Bianca Ambrogina Silva, Fabrizio Gardoni, Monica DiLuca, Diego Scheggia

**Affiliations:** 1 Department of Pharmacological and Biomolecular Sciences “Rodolfo Paoletti”, University of Milan, Milan, Italy; 2 Université Côte d’Azur, CNRS UMR7275, INSERM U1318, Institut de Pharmacologie Moléculaire et Cellulaire, Valbonne, France; 3 Institute of Neuroscience, National Research Council of Italy, Milan, Italy; 4 Lab of Circuits Neuroscience, IRCCS Humanitas Research Hospital, Milan, Italy

**Keywords:** Social behaviour, Neural circuits

## Abstract

Animals can learn about danger by observing conspecifics, but whether and how they acquire behaviors through positive affective states of others is not understood. Here we show that by observing a demonstrator, mice learn to take actions that benefit others and that are goal-directed and flexible. Chemogenetic silencing experiments showed that activity in hippocampal dorsal CA1 (dCA1) was required for observers to learn action–outcome associations in a social context. Fiber photometry recordings revealed inter-individual differences in dCA1 activity patterns during observation that tracked the observer’s subsequent prosocial or selfish behavioral propensities. Optogenetic manipulations demonstrated that the dCA1 is key during observation and can orient a mouse’s actions toward prosocial or selfish choices in future interactions. Our study provides a mouse model of social transmission of knowledge that guides prosocial behavior and that may be relevant for studying disorders in which the ability to learn from others’ actions is compromised.

## Abstract (web summary)

La Greca and colleagues show that by witnessing a conspecific’s actions, mice learn how actions can lead to shared food, forming flexible social action–outcome associations that require dorsal CA1 and bias future prosocial or selfish behaviors.

## Main

The ability to share is a foundation of human evolution and represents a notable facet of prosocial behaviors. Humans and animals often share resources and help others, even when there are no immediate benefits for themselves^1–3^. Exploiting other individuals’ knowledge through observation can lead to acquiring socially relevant behaviors, thereby increasing social integration and cooperation^4^. Observational learning is a fundamental trait in many species^5–7^. Like humans, animals can learn to associate aversive stimuli and cues that predict danger from others^6,8,9^, reducing the risks associated with direct experience^10^. Observational learning can also involve an affective empathic response to environment adaptation. Mice can detect and share the affective state of others^6,11–13^, which in turn can favor different forms of prosocial behaviors, such as consolation^14^, helping^15^, harm avoidance^16^ and providing rewards^17,18^.

Observational fear learning, where threats are learned through observation, recruits the cortical areas^9,11,19,20^ and basolateral amygdala (BLA)^6,11^. Furthermore, understanding others’ aversive situations recruits hippocampal functions to construct and remember contextual experience, which can guide future behaviors^8^; however, little information is available on the neurobiology of learning that promotes behaviors with positive valence through socially transmitted information.

Here, we used a social decision-making (SDM) paradigm in mice in which observers (OBS) can learn from demonstrators (DEM) how to share a food reward. Through observation, mice not only learned the nose poke–outcome association but also made more coherent choices than after classical trial-and-error learning. Furthermore, mice displayed an increased propensity to select actions resulting in food sharing within the social context, a behavior shaped by individual variability, familiarity and sharing behavior of the DEM. We identified neuronal activity- and plasticity-dependent changes in the dorsal hippocampal CA1 (dCA1) following observation of prosocial behavior. Chemogenetic and optogenetic manipulations revealed that the dCA1 is a determinant for learning associations between actions and outcomes in a social context during observation—associations that subsequently guide future choices. Our study demonstrates that the dCA1 has a critical role in learning socially transmitted information that can drive prosocial behaviors.

## Results

### Mice learn decision-making by observation

**Fig. 1:**
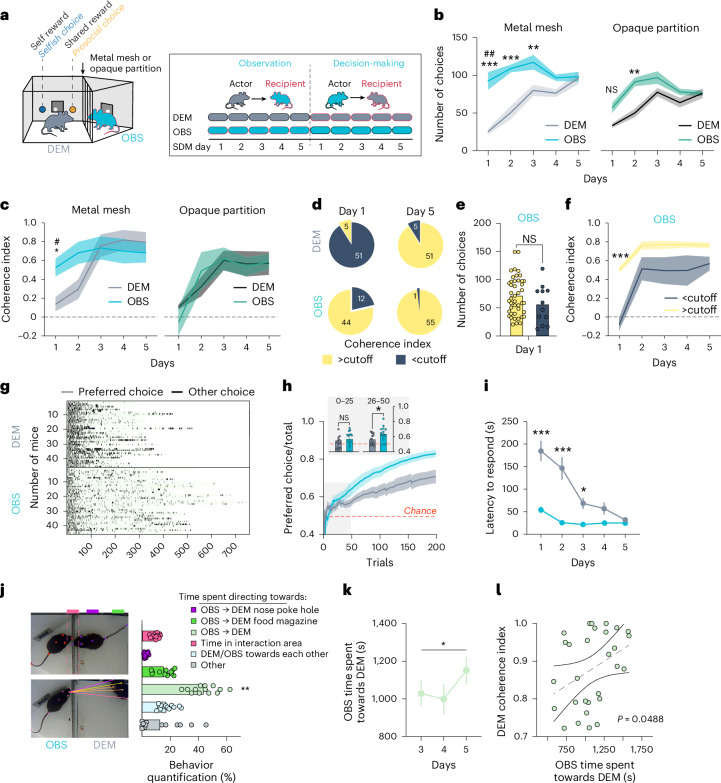
Mice learn decision-making by observation. **a**, Experimental setup. **b**,**c**, Number of choices (**b**, two-way repeated measures (RM) analysis of variance (ANOVA), time (days) × group (DEM, OBS, metal mesh, opaque partition), *F*_(12,96)_ = 4,71, *P* < 0.0001) and coherence index (**c**, time × group, *F*_(12,96)_ = 2,28, *P* = 0.0133) of DEM and OBS tested with a metal mesh (left, *n* = 7 dyads) or an opaque partition (right, *n* = 7 dyads). *P < 0.05, **P < 0.01, ***P < 0.005 versus DEM, same condition; ^#^*P* < 0.05, ^##^*P* < 0.01 versus OBS, opaque partition. **d**, Pie charts showing the proportions of DEM and OBS below (<cutoff) and above (>cutoff) the coherence index cutoff set to 0.20 on day 1 (left, Fisher’s exact test, *P* < 0.0001) and day 5 (right, *P* = 0.206). Data (**d**–**i**) were obtained from 14 independent experiments. **e**,**f**, Number of choices on day 1 (**e**, two-tailed, unpaired *t*-test, *t* = 1,404, d.f. = 54, *P* = 0.1661) and coherence index across 5 days of decision-making phase (**f**, two-way RM ANOVA, time (days) × group (OBS <cutoff, >cutoff), *F*_(4,216)_ = 3,74, *P* = 0.0057) of OBS below (<cutoff *n* = 12) and above (>cutoff *n* = 44) the coherence cutoff. NS, not significant. **g**, Heatmap showing mice preferred (white) or other choice (black) across the 5 days of SDM. Each raw represents data from one DEM or OBS. **h**, Trial-by-trial preferred nose poke choice over total number of nose pokes (*n* = 56 DEM–OBS dyads) and during nose pokes 0 to 25 (inset, two-tailed, unpaired *t*-test, *t* = 1,385, d.f. = 28, *P* = 0.1770) and 26 to 50 (*t* = 2,265, d.f. = 28, *P* = 0.0314). **i**, Latency to respond (time between trial start and nose poke) of DEM and OBS in the SDM (two-way RM ANOVA, time (days) × group (DEM, OBS), *F*_(4,328)_ = 16.3, *P* < 0.0001, *n* = 56 DEM–OBS dyads). **j**, Representative images of pose estimation model in DeepLabCut (top) and SimBA analysis for quantification of time directing toward DEM (bottom, one-way ANOVA, *F*_(1.141, 13.69)_ = 33,20, *P* < 0.0001, *n* = 13 OBS). **k**, OBS time spent directed toward DEM increases over testing days (one-way RM ANOVA, *F*_(1.740, 20.88)_ = 33.20, *P* = 0.0225, *n* = 13 OBS). **l**, Correlation between DEM coherence index and OBS time spent directing toward DEM (linear regression, *r*^2^ = 0.06999). Error bars and shading represent s.e.m. unless otherwise noted. *P < 0.05; **P < 0.01; ***P < 0.001; NS, not significant.

To test whether mice learn how to make decisions by observing others we used the SDM paradigm that assesses inter-individual propensities for sharing a food reward in mice^18^. In an expanded operant cage, decision-maker mice, acting as ‘demonstrators’ (DEM), using nose poke holes, decided to take a food reward only for themselves (hereafter labeled as a ‘selfish choice’) or to share the reward (‘prosocial choice’) with recipient mice, acting as ‘observers’ (OBS; Fig. 1a). The OBS were in an adjacent compartment, separated by a metal mesh, and could receive food rewards only upon DEM choices. After 5 days in this compartment (‘observation’; Fig. 1a), the OBS replaced their DEM and operated as decision-makers. The OBS were allowed to act as decision-makers only after the observation period to avoid the ‘mere presence’ effect^21^. In this phase (‘decision-making’; Fig. 1a), we assessed whether mice learned from previous observations of their partners. We compared this group to a control group of mice tested with an opaque partition that allowed auditory and olfactory but not visual stimuli.

We found that OBS made more nose pokes than their DEM and OBS tested with the opaque partition (Fig. 1b), indicating that observation improved the response–outcome association. Furthermore, we quantified the ability of the mice to choose and discriminate between the two options, calculating a coherence index, where positive values indicate consistent choices across days. We set a cutoff to distinguish mice that displayed a clear (>60% of responses into one of the two nose pokes) and coherent (choosing the same nose poke across sessions) preference throughout the decision-making phase. We found that OBS had a higher coherence index on day 1 than their DEM and the OBS tested with the opaque partition (Fig. 1c), suggesting they had learned to perform the task during the observation phase. In contrast, OBS tested with the opaque partition had a coherence index close to zero, indicating that they performed at chance level (Fig. 1c).

**Extended Data Fig. 1:**
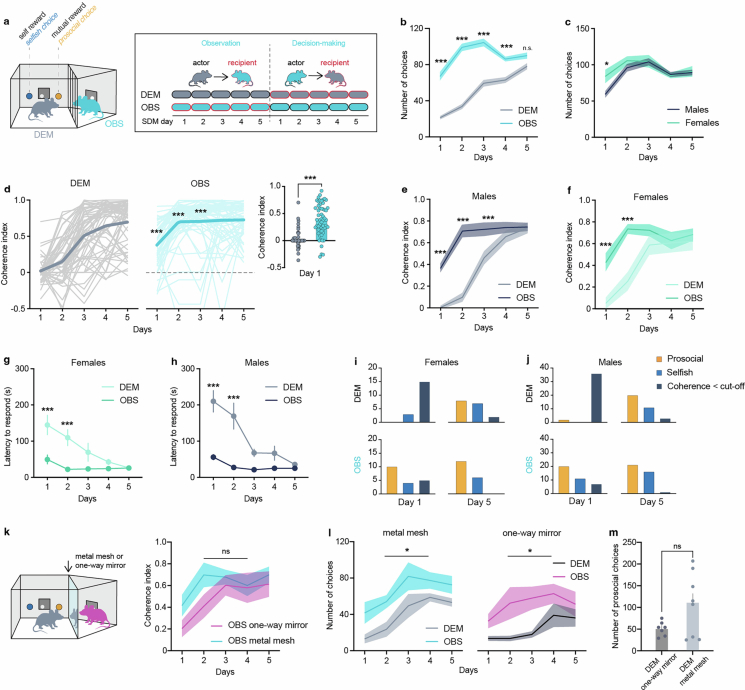
Female and male mice learn social decisions through others’ observation. **a**, Experimental setup. **b**, **c**, Mean number of choices across days in the SDM of DEM and OBS (**b**, two-way RM ANOVA, time (days) x group (DEM, OBS), F_(4,440)_ = 32.59, *P* < 0.0001, *n* = *56* dyads) and OBS grouped by sex (**c**, time (days) x group (males, females), F_(4,216)_ = 2.72, *P* = 0.0308, males *n* = *38*, females *n* = *18*). **d**, Individual curves of coherence index of DEM and OBS across days in the SDM (two-way RM ANOVA, time (days) x group (DEM, OBS), F_(4,440)_ = 24.2, *P* < 0.0001, *n* = *56* dyads). Bold curve represents group mean. *Inset*, average coherence index of DEM and OBS on day 1 in the SDM (two-tailed unpaired t-test, t = 8.471, df = 110, *P* < 0.0001). **e**, **f**, Coherence index across days in the SDM of DEM and OBS grouped by males (two-way RM ANOVA, time (days) x group (DEM, OBS), F_(4,296)_ = 18.2, *P* < 0.0001, *n* = *38* dyads) and females (F_(4,136)_ = 7.01, *P* < 0.0001, *n* = *18* dyads). **g**, **h**, Latency to respond (time between trial start and nose poke) of females (**g**, two-way RM ANOVA, time (days) x group (DEM, OBS), F_(4,96)_ = 6.05, *P* = 0.0002, *n* = 14 DEM*, n* = 12 OBS dyads) and males (**h**, F_(4,200)_ = 11.03, *P* < 0.0001, *n* = 22 DEM*, n* = 30 OBS) DEM and OBS across days in the SDM. **i**, **j**, Number of female (**i**) and male (**j**) DEM (*top*) and OBS (*bottom*) with preference for prosocial (*orange*) or selfish choice (*blue*) or with coherence index below the cut-off (*dark gray*). **k**, Coherence index in the decision-making phase of the SDM of OBS mice tested with the metal mesh (*n* = 8) or a one-way mirror (*n* = *7;* two-way RM ANOVA, time (days) x group (OBS, metal mesh, one-way mirror), F_(4,52)_ = 1,011, *P* = 0.4106*)*. **l**, Mean number of choices across days in the SDM of DEM and OBS tested with a metal mesh (*left*) or a one-way mirror (*right*, two-way RM ANOVA, time (days) x group (DEM and OBS metal mesh, DEM and OBS one-way mirror), F_(12,104)_ = 2.00, *P* = 0.0306). *P < 0.05 DEM vs OBS. Data shown as mean ± s.e.m. *P < 0.05, ***P < 0.001. NS not significant. # compared to OBS opaque partition. **m**, Total number of prosocial choices made by DEM tested with metal mesh or a one-way mirror (two-tailed unpaired t-test, t = 2.083, df = 7.70, *P* = 0.0719, DEM with the metal mesh *n* = 8, DEM one-way mirror *n* = *7*). Error bars and shading represent the s.e.m. unless otherwise noted.

We replicated the SDM several times (*n* = 56 DEM–OBS dyads), in males and females, in naive and control virus-injected mice (for later manipulations) and confirmed an increased number of choices (Extended Data Fig. 1b) and coherence index (Extended Data Fig. 1d) in the OBS compared to their DEM. Similar results were obtained in male and female OBS (Extended Data Fig. 1c–f). Only 5 of the 56 DEM showed a preference on day 1 that was maintained throughout the task (Fig. 1d); most performed at chance level on day 1 (coherence index <0.20), only reaching a stable performance in later daily sessions (Fig. 1d). In contrast, most OBS (44 of the 56) already displayed consistent choices (coherence index >0.20) on day 1 (Fig. 1d).

**Extended Data Fig. 2:**
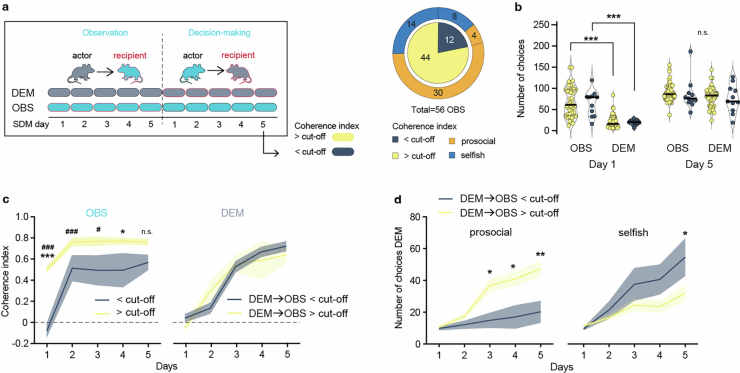
Observational learning stratified by coherence index and associated demonstrator behavior. **a**, Total number of OBS grouped by coherence index cut-off (dark gray < cut-off; yellow, > 0.20) by preference (blue, selfish; orange, prosocial). **b**, total number of choices made by OBS grouped by coherence index and their DEM on day 1 and 5 of the SDM (two-way RM ANOVA, time (days) x group (DEM, OBS, < cut-off, > cut-off), F_(3,108)_ = 10.35, *P* < 0.0001, *n* = 44 > cut-off, *n* = 12 < cut-off). Violin plots display the distribution of individual data points, with the bold line indicating the median and the thinner lines representing the 25th and 75th percentiles. **c**, Coherence index of OBS (*left*) above (*n* = 44) or below (*n* = 12) the cut-off and (*right*) coherence index of their DEM (three-way RM ANOVA, group (DEM, OBS) x cut-off (above,below), F_(1,108)_ = 8.04, *P* = 0.0055, *n* = 44 > cut-off, *n* = 12 < cut-off). **d**, Prosocial and selfish choices of DEM paired with OBS above or below the cut-off (two-way RM ANOVA, time (days) x group (DEM → OBS < cut-off, DEM → OBS > cut-off), F_(4,216)_ = 4.63, *P* = 0.0013, *n* = 44 > cut-off, *n* = 12 < cut-off). *P < 0.05, **P < 0.01, ***P < 0.001. ^###^*P* < 0.001 vs. DEM. NS, not significant. Error bars and shading represent the s.e.m. unless otherwise noted.

We separately examined OBS with coherence indexes above or below the cutoff (Fig. 1e,f and Extended Data Fig. 2a). Mice from both OBS groups showed a similar number of choices on day 1 (Fig. 1e), which were increased compared to their DEM (Extended Data Fig. 2b), indicating that they had acquired the response–outcome association; however, the OBS group with coherence indexes below the cutoff chose randomly between nose pokes or showed incoherent preferences on subsequent days (Fig. 1f). These results indicate that mice developed a preference for one of the two choices during the observation phase and learned how to display it, without the need for initial training, in the decision-making phase.

Analyzing trial-by-trial responses, we found that OBS chose the less preferred nose poke fewer times than their DEM (Fig. 1g). On day 1 both groups began at chance, but OBS converged on the preferred response within 25 trials (Fig. 1h). Furthermore, we measured the response latency, which is the time between two consecutive choices. We found that OBS had shorter latencies than DEM throughout testing (Fig. 1i), which is a further indication of learning.

Finally, we examined OBS postural dynamics during the SDM observation phase to categorize other behaviors. The OBS were tracked using DeepLabCut^22^, and their behaviors were classified with SimBA^23^ (Fig. 1j). By inspecting postures, positions and movements, we annotated how much time OBS spent directing toward the DEM (facing DEM, nose-to-nose interaction and facing each other) or toward stationary objects (DEM nose poke holes and food magazine) (Fig. 1j). Most of the time, OBS were directed toward their DEM. Of note, the time spent directed toward their DEM increased across days (Fig. 1k) and correlated positively with the coherence index of their DEM (Fig. 1l), suggesting that OBS were more focused on the DEM’s ability to make coherent choices.

We then asked whether social contact and reciprocal interaction were necessary for observational learning. To address this point, we tested OBS with a one-way mirrored divider allowing OBS to witness DEM behavior, while preventing DEM from seeing OBS and compared to OBS tested with the standard metal mesh divider allowing full bidirectional interaction (Extended Data Fig. 1k). OBS in both groups were then tested in the decision-making with the metal mesh. We found no differences between OBS that were tested in the observation phase with the one-way mirror or the metal mesh (Extended Data Fig. 1l,m). These results indicate that observational learning in this paradigm occurs independently of reciprocal social interaction and is primarily driven by the OBS ability to witness the DEM actions and outcomes, rather than by receiving feedback from the DEM.

### Observational learning is not a manifestation of mimicry

**Extended Data Fig. 3:**
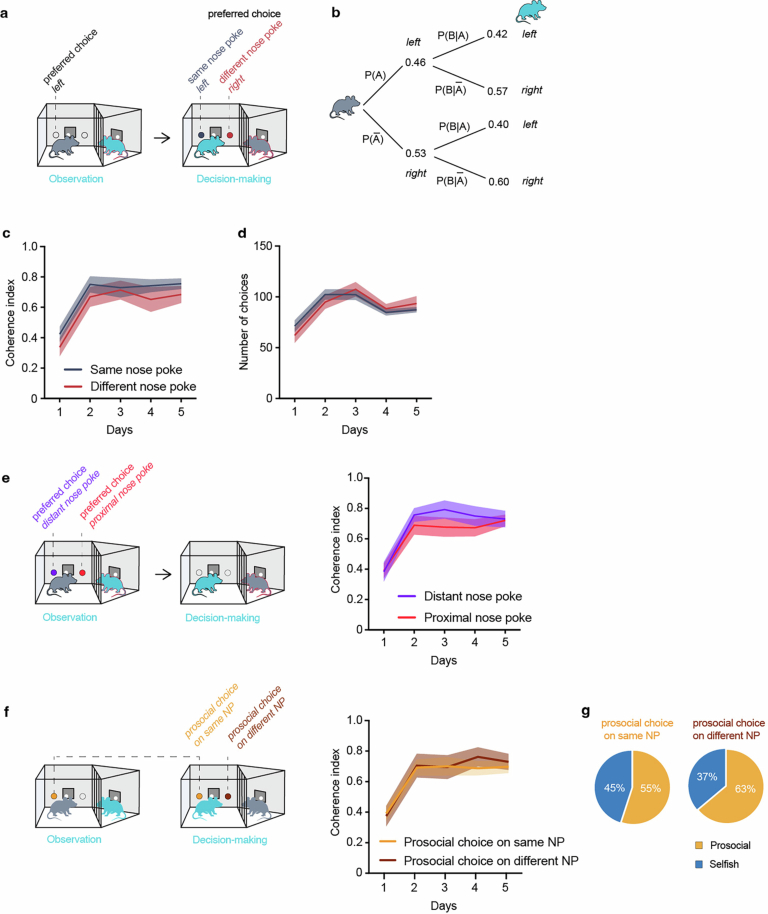
Observational learning is not driven by side-specific imitation and generalizes across nose poke configurations. **a**, Schematic of the SDM. OBS mice were grouped by preference for the same nose poke (same location) preferred by the DEM or for the opposite nose poke (different location). **b**, Probability tree showing the occurrence of OBS preference for the same or different nose poke location chosen by their DEM (two-sided Fisher’s exact test > 0.99). **c**, **d**, Coherence index (**c**, two-way RM ANOVA, time (days) x group (same,different location), F_(4,216)_ = 0.23, *P* = 0.9175), mean number of choices (**d**, F_(4,216)_ = 1.37, *P* = 0.2441), in OBS mice grouped by preference for nose poke location (same location, *n* = 33, different location, *n* = 23). **e**, Coherence index of OBS showing preference for left (*distant location, n* = *22*) or right (*proximal location, n* = *34*) nose poke hole (two-way RM ANOVA, time (days) x group (distant, proximal location). **f**, **g**, Coherence index (**f**, two-way RM ANOVA, time (days) x group (same, different), F_(4,212)_ = 0.32, *P* = 0.8604) and pie charts (**g**, two-sided chi-square test, 0.42, df = 1, *P* = 0.5140) for prosocial (orange) or selfish (blue) preference of OBS presented during the decision-making phase with prosocial choice associated with the same nose poke as DEM (*same, n* = *36*) or on a different nose poke (*different, n* = *20*). Error bars and shading represent the s.e.m.

We then asked whether OBS learning was merely imitating DEM behavior. To answer this question, we checked whether OBS chose the same nose poke location preferred by their DEM in the SDM (Extended Data Fig. 3a). Using a probability tree, we found that OBS did not necessarily prefer the nose poke location chosen by their DEM (Extended Data Fig. 3b), suggesting that OBS were not strictly anchored to the same sequence of actions witnessed during the observation phase. Furthermore, OBS displaying a preference for the same or different nose poke location did not differ in their coherence indexes (Extended Data Fig. 3c,d).

We also checked whether learning from DEM preferring nose poking into a proximal or distant nose poke could have influenced OBS learning. However, we found no differences in the coherence index (Extended Data Fig. 3e), excluding the possibility of bias due to place preference. Next, to assess the flexibility OBS have when applying the observed behavior in a modified context, in the decision-making phase we presented to a subgroup of OBS (20 of the 56) a nose poke configuration that differed from the one used during the observation phase (Extended Data Fig. 3f). Under this condition, we did not report significant differences from the OBS presented with the same nose poke configuration of the DEM (Extended Data Fig. 3f,g). Altogether, these results indicate that OBS were sufficiently flexible to adapt the observed behavior to a modified context.

### Observers learn socially mediated action–reward association

**Fig. 2:**
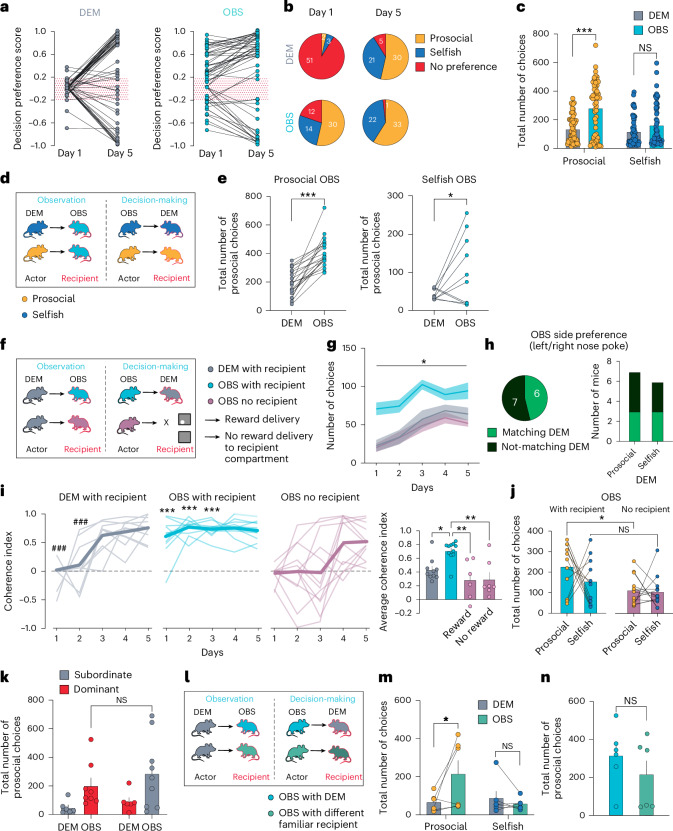
Observers learn socially mediated action–reward association. **a**, Decision preference score of DEM (left, *n* = 56) and OBS (right, *n* = 56) on day 1 and 5. **b**, Pie charts showing the proportions of DEM and OBS displaying preference for prosocial (orange) or selfish (blue) choice or displaying no preference (red), on day 1 (left, chi-squared test, 58.31, d.f. = 2, *P* < 0.0001) and day 5 (right, chi-squared test, 28.33, d.f. = 2, *P* = 0.2426). **c**, Total number of prosocial and selfish choices by DEM and OBS during their decision-making phase of the SDM (two-way ANOVA, choice (prosocial, selfish) × group (OBS, DEM), *F*_(1,110)_ = 4.378, *P* = 0.0387, *n* = 56 dyads). **d**, Decision-making phase of OBS paired to DEM displaying same preference. **e**, Total number of prosocial choices made by prosocial OBS compared to their DEM (left, two-tailed, unpaired *t*-test, *t* = 6.787, d.f. = 38, *P* < 0.0001, *n* = 20 dyads) and by selfish OBS and their DEM (right, *t* = 2.105, d.f. = 18, *P* = 0.0496, *n* = 10 dyads). **f**, Following observation, OBS were tested in the decision-making phase with their original DEM (acting as recipient) or without recipient in the adjacent compartment. **g**, Number of choices (**g**, two-way RM ANOVA, group (DEM with recipient, OBS with recipient, OBS no recipient), *F*_(2,31)_ = 13.37, *P* < 0.0001). **h**, Pie chart (left) showing the proportion of OBS matching (light green) or not-matching (dark green) the side preference of their DEM and graph (right) showing relation to DEM prosocial or selfish preference (Fisher’s exact test, *P* > 0.9999). **i**, Coherence index (left, time × group, *F*_(8,124)_ = 5.05, *P* < 0.0001) of DEM (*n* = 10) and OBS tested with a recipient (*n* = 11) and OBS tested with no recipient (*n* = 13). ***P < 0.005 OBS with recipient versus OBS no recipient; ^###^*P* < 0.0015 DEM versus OBS no recipient. Right: average coherence index across days 1–5 *(*one-way ANOVA, *F*_(3,30)_ = 8.311, *P* = 0.0004). **j**, Total number of prosocial and selfish choices by OBS tested with a recipient (*n* = 10) and with no recipient (*n* = 10) (two-way ANOVA, group (with recipient, no recipient), *F*_(1,22)_ = 29.18, *P* < 0.0001). **k**, Total number of prosocial choices by DEM (*n* = 6–8) and OBS (*n* = 8–9) grouped by social rank (subordinate, gray/dominant, red; two-way ANOVA, rank (subordinate/dominant) *F*_(1,25)_ = 1.056, *P* = 0.3140, group (OBS, DEM), *F*_(1,25)_ = 7.669, *P* = 0.0104; rank × group, *F*_(1,25)_ = 0.120, *P* = 0.7310). **l**, Following observation, OBS were tested in the decision-making phase with their original DEM (acting as recipient) or a different, familiar, recipient. **m**, Total number of prosocial and selfish choices by DEM and OBS during their decision-making phase of the SDM (two-way ANOVA, choice (prosocial, selfish) × group (OBS, DEM), *F*_(1,10)_ = 4.95, *P* = 0.050, DEM *n* = 6, OBS with different familiar recipient *n* = 6). **n**, Total number of prosocial choices in the SDM by OBS with DEM (*n* = 6) and OBS with different familiar recipient (*n* = 6; two-tailed, unpaired *t*-test, *t* = 0.8709, d.f. = 5, *P* = 0.4236). Data shown as mean ± s.e.m. *P < 0.05; ***P < 0.001; NS, not significant.

Based on these results, we next examined the preference of the DEM and OBS for prosocial or selfish choices. We previously reported that most mice preferred to share food rewards in the same task^18^. A similar preference was displayed by the DEM (30 preferred prosocial choices), as indicated by positive decision preference scores (Fig. 2a,b). Similarly, most OBS displayed a preference for prosocial over selfish choices. However, unlike their DEM, this preference was already evident on day 1 of testing and remained stable until the end (Fig. 2a,b and Extended Data Fig. 1i,j).

Consistent with the above finding that OBS made more choices than DEM (Fig. 1b), we found, at the group level, that OBS made more prosocial choices (Fig. 2c). This effect was specific to prosocial choices, as their selfish choices were unchanged compared to their DEM (Fig. 2c). Notably, not only prosocial OBS (Fig. 2d) but also selfish OBS (Fig. 2e) made more prosocial choices than their DEM.

As OBS receive rewards only when DEM make prosocial choices during the observation, their subsequent preference could reflect learning a side bias, rather than the association between observed action and its social outcome. To address this, we tested OBS in their decision-making without a recipient (Fig. 2f) and found a decreased number of nose pokes compared to OBS tested with a recipient (Fig. 2g). We compared the nose poke side preference of OBS tested with no recipient to that of their respective DEM. Of the 13 OBS in this condition, 6 matched their DEM preferred side, whereas 7 chose the opposite (Fig. 2h). Additionally, whether the DEM preferred prosocial or selfish nose pokes did not significantly influence this matching outcome (Fig. 2h). OBS with no recipient also showed inconsistent choice patterns across sessions compared to OBS tested with recipient as well as to DEM group (Fig. 2i). Furthermore, reward delivery to the empty recipient compartment did not modify the behavior (Fig. 2i). Finally, OBS with no recipient made fewer prosocial choices compared to those with a recipient, whereas selfish choices remained unchanged (Fig. 2j). Altogether, these data indicate that OBS choices were not driven by side bias or sensory cues associated with reward delivery, rather for the presence of the rewarded partner. Because removing the recipient modifies the experienced action–outcome contingency, the reduced prosocial bias in this condition might also reflect updating of learned associations.

Social hierarchy among cage mates could influence prosocial behavior^24^. We thus checked whether the increase in prosocial choices was the result of observational learning or was determined by social dominance. We assessed the social rank of each mouse using the tube test and then OBS mice were paired to dominant or subordinate DEM in the SDM task. We found that both groups of mice showed increased prosocial choices compared to DEM, but we did not detect any differences between dominant and subordinate OBS (Fig. 2k). This result confirmed that observed prosocial actions could promote prosocial behavior in future interactions.

Next, to determine whether the increase in OBS prosocial choices was explained by an act of reciprocity with their DEM we tested OBS with a different familiar partner mouse (another cagemate; Fig. 2l). We found that, similarly to OBS tested with their DEM, OBS mice showed an increased number of prosocial choices also when tested with a different familiar partner (Fig. 2m). Furthermore, there was no difference in the number of prosocial choices between OBS tested with their DEM or with the different familiar recipient (Fig. 2n). These findings indicate that OBS flexibly generalize their learned behavior to new but familiar social contexts.

**Extended Data Fig. 4:**
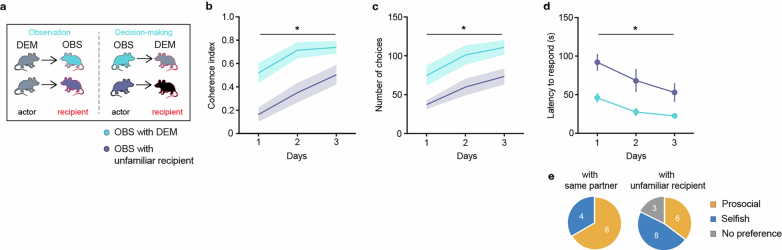
Familiarity of the recipient modulates expression of socially acquired action–outcome associations. **a**, OBS mice following observation with a familiar DEM (a cagemate) were tested in the decision-making phase with their DEM or with an unfamiliar recipient. **b**–**d**, Coherence index (**b**, two-way RM ANOVA, Group (same partner, different partner), F_(1,27)_ = 13.1, *P* = 0.0012), number of choices (**c**, F_(1,27)_ = 9.36, *P* = 0.0050) and latency to respond (**d**, F_(1,27)_ = 11.0, *P* = 0.0026) of OBS tested in the decision-making phase of the SDM with the same partner (previous DEM, *n* = 12) or with a different partner (unfamiliar mouse, *n* = 17). **e**, Pie charts showing the number of OBS with same or different partner showing preference for prosocial (orange) or selfish (blue) choices or with no preference (gray) (chi-square test, 3.87, df = 2, *P* = 0.1443). Error bars and shading represent the s.e.m.

Finally, we tested a group of mice that, after the observation phase with their DEM, were exposed to an unfamiliar partner during the decision-making phase (Extended Data Fig. 4a). In this case we found that the OBS with a novel recipient displayed a lower number of choices compared to OBS that remained with their DEM (Extended Data Fig. 4c). Moreover, OBS tested with an unfamiliar recipient displayed more selfish preference (Extended Data Fig. 4e), consistent with the role of familiarity in enhancing prosocial behaviors^18,25^.

### Individual differences in observational learning

**Fig. 3:**
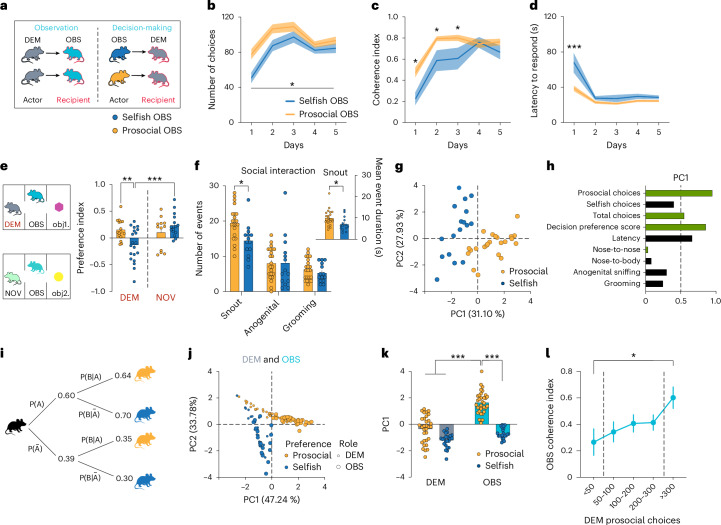
Individual differences in observational learning. **a**, Following the observation phase mice behaviors in the decision-making phase were analyzed separately based on their preference displayed for prosocial or selfish choices. **b**,**c**, Number of choices (**b**, two-way RM ANOVA, group (prosocial OBS, selfish OBS), *F*_(1,54)_ = 8.434, *P* = 0.0053; time (days), *F*_(4,216)_ = 20.61, *P* < 0.0001) and coherence index (**c**, time (days) × group (prosocial OBS, selfish OBS), *F*_(4,216)_ = 3.00, *P* = 0.0194) of prosocial (*n* = 34) and selfish (*n* = 22) OBS in the decision-making phase of the SDM. **d**, Latency to respond of prosocial (*n* = 27) and selfish (*n* = 17) OBS (two-way RM ANOVA, time (days) × group (prosocial OBS, selfish OBS), *F*_(4,168)_ = 4.91, *P* = 0.0009). **e**, Preference index of OBS mice in the three-chamber test with their DEM (prosocial *n* = 16, selfish *n* = 20) or with a novel unfamiliar mouse (prosocial *n* = 12, selfish *n* = 16) (two-way ANOVA, group (prosocial OBS, selfish OBS) × stimulus (DEM, novel), *F*_(1,60)_ = 10.28, *P* = 0.0022). **f**, Number of events (grouped by snout and anogenital) and self-grooming behavior during free social interaction tested in a standard cage between prosocial (*n* = 22) and selfish (*n* = 22) OBS with their DEM (two-way RM ANOVA, behavior × group (prosocial and selfish OBS), *F*_(2,68)_ = 3.24, *P* = 0.0453). Inset: mean event duration for nose-to-nose sniffing (two-tailed, unpaired *t*-test, *t* = 2.382, d.f. = 34, *P* = 0.0230). **g**, PCA of the behavioral profile of OBS mice considering all the variables from **b**–**f** (*n* = 40). **h**, Top contributing behaviors of PC1 (black and green represent negative and positive values). **i**, Tree diagram of the conditional probability of OBS mice displaying prosocial or selfish preference following learning with prosocial or selfish DEM (Fisher’s exact test, *P* = 0.7672). **j**, PCA of the behavioral measurements of OBS and their DEM during the SDM (*n* = 112). **k**, PC1 scores of DEM (*n* = 56) and OBS (*n* = 56) grouped by preference for prosocial and selfish choices (two-way ANOVA, group (DEM, OBS) × preference (prosocial, selfish), *F*_(1,108)_ = 24.56, *P* < 0.0001). **l**, OBS coherence index on day 1 of the decision-making phase of the SDM in relation to the number of their DEM prosocial responses (*n* = 56 dyads, two-tailed unpaired *t*-test (<50, >300), *t* = 2.609, d.f. = 13.8, *P* = 0.0208). Data are shown as mean ± s.e.m. *P < 0.05; **P < 0.01; ***P < 0.001; NS, not significant.

We next investigated how individual differences in social preferences manifested following observation. We separately examined the OBS based on their displayed preferences during the decision-making phase to determine whether prosocial and selfish tendencies were associated with distinct learning patterns (Fig. 3a). We found that prosocial OBS made more choices than selfish OBS (Fig. 3b). The choices of prosocial OBS were more focused, as demonstrated by a higher coherence index (Fig. 3c) and reduced time between choices (Fig. 3d) than selfish OBS.

Building upon these findings, to confirm that OBS learned the social contingencies of the observational conditioning, we tested whether OBS preference for prosocial or selfish choices could be transferred to other contexts. Following the SDM, the OBS were tested in a three-chamber arena, in which their DEM and an inanimate object were placed on opposite sides (Fig. 3e). In this experiment, prosocial OBS preferred their DEM over the object, whereas selfish OBS preferred the object (Fig. 3e). When tested with a novel mouse, selfish OBS preferred the partner over the object, indicating intact sociability (Fig. 3e). Then, we tested a different subgroup of OBS in a 10-min free social interaction in a standard plastic arena, where they were allowed to interact with their DEM partner (Fig. 3f). We found that the prosocial OBS engaged in more and longer direct nose-to-nose contacts with their DEM than the selfish OBS (Fig. 3f), confirming the previous observation in this experimental setting.

### Prosocial actions favor observational learning

**Extended Data Fig. 5:**
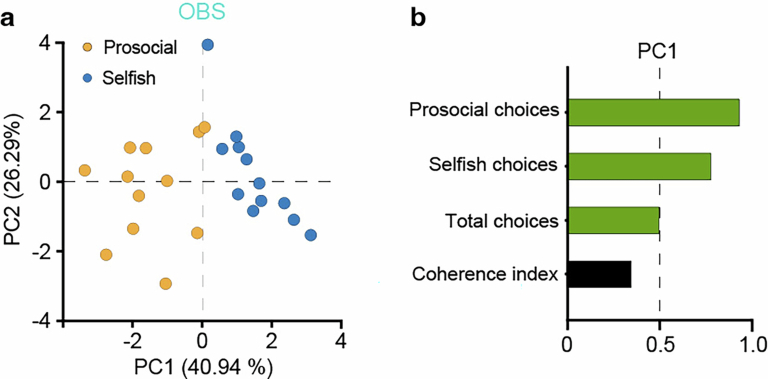
Principal component analysis of behavioral variables in observers. **a**, Principal component analysis (PCA) of the behavioral profile of OBS considering all the variables from Fig. 3 panels **b**–**g** (*n* = 24). **b**, top contributing behaviors of PC1 (black and green represent negative and positive values respectively).

To evaluate the contribution of all recorded behavioral parameters and determine behavioral patterns related to OBS displaying prosocial or selfish preferences, we performed dimensionality reduction using principal-component analysis (PCA) in two different cohorts of mice. PCA incorporating all recorded behavioral parameters identified three principal components (PCs) explaining a substantial percentage of the total variance (cohort 1, 76.30%, Fig. 3g; cohort 2, 83.97%, Extended Data Fig. 5a). Notably, PCA clearly distinguished prosocial from selfish OBS in both cohorts (Fig. 3g and Extended Data Fig. 5a), and we found a significant difference in PC1 values between prosocial and selfish OBS. The major variable contributing to PC1 was the number of prosocial choices (Fig. 3h).

To understand whether a prosocial or selfish DEM could influence the future choices of OBS we calculated the probability of OBS displaying a preference for prosocial choices following observing a prosocial or selfish DEM; however, this probability did not change (Fig. 3i).

We then asked whether, more specifically, prosocial choices could influence OBS learning. To answer this question, we performed the PCA to identify DEM and OBS using the parameters measured in the SDM (Fig. 3j). The DEM and OBS differed in the PC1, which also, in this case, was mainly guided by the number of prosocial choices (Extended Data Fig. 5b). We then stratified DEM and OBS based on their prosocial and selfish preferences, revealing a significant difference in PC1 values between prosocial OBS and DEM (Fig. 3k). Therefore, we hypothesized that the number of prosocial choices made by the DEM could positively influence OBS learning. Indeed, we found that the OBS coherence index correlated positively with the number of prosocial choices, but not selfish choices, made by the DEM during the observation phase (Fig. 3l). This observation is also supported by the findings showing that the DEM of OBS with a coherence index above the cutoff made more prosocial choices than the DEM of OBS with a coherence index below the cutoff (Extended Data Fig. 2d). Therefore, while overall DEM preference does not simply determine OBS behavior, the experience of shared rewards during observation could play a key role in shaping future choices.

### dCA1 is engaged during observed prosocial behaviors

**Fig. 4:**
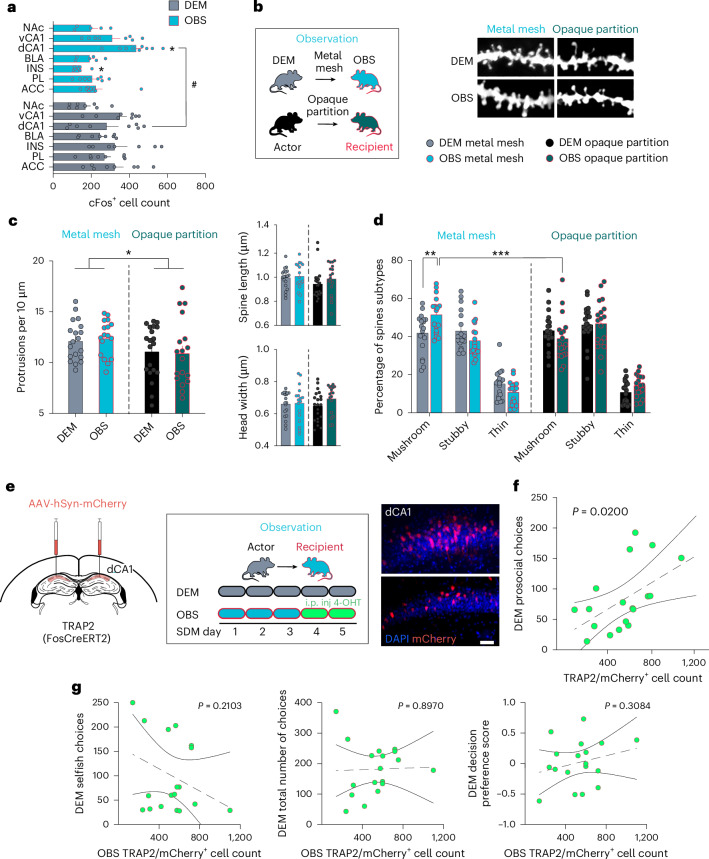
dCA1 is engaged during observed prosocial behaviors. **a**, Left: quantification of cFos+ cells across seven brain regions (at least three sections per region) in DEM (*n* = 8) and OBS (*n* = 9) (two-way ANOVA, brain region × group (DEM, OBS), *F*_(6,93)_ = 3.65, *P* = 0.0026). *P < 0.05 versus DEM; ^#^*P* < 0.05 versus all other regions of OBS. **b**, Left: timeline of dCA1 neuron morphology analysis in DEM and OBS tested with a metal mesh or an opaque partition during the observation phase of the SDM. Right: representative images showing dendrites of OBS and DEM in dCA1. **c**,**d**, Protrusion densities (left, two-way ANOVA, condition (metal mesh, opaque partition), *F*_(1,70)_ = 0.15, *P* = 0.0335) and spine length (right, *F*_(1,71)_ = 1.89, *P* = 0.1725) and head width (*F*_(1,71)_ = 0.81, *P* = 0.7220) of dendritic spines (**c**) and proportions of different spine types in percentage of total spines (mushroom, stubby and thin, three-way ANOVA, group (DEM, OBS) × condition × spine type, *F*_(2,213)_ = 7.62, *P* = 0.0006) in dCA1 of mice tested with a metal mesh (DEM *n* = 19 and OBS *n* = 17 neurons, from four mice) or an opaque partition (DEM *n* = 20 and OBS *n* = 18 neurons, from four mice) during the observation phase of the SDM (**d**). **e**, Timeline to ‘TRAP’-activated neurons during the observation phase of the SDM and representative photomicrographs of the area for mCherry^+^ TRAPed cells count in the dCA1 of OBS paired to DEM with positive (top) or negative (bottom) decision preference score. DAPI, 4′,6-diamidino-2-phenylindole. Scale bar, 50 μm. **f**, Correlation between TRAP2/mCherry^+^ cells following the observation of the SDM and number of their DEM prosocial responses (linear regression, *n* = 18). **g**, Correlation between TRAP2/mCherry^+^ cells following the observation of the SDM and number of DEM selfish choices (left), DEM total number of choices (middle) and DEM decision preference score (right) (linear regression, *n* = 18). Error bars and shading represent the s.e.m. unless otherwise noted.

We examined the brain regions activated by observational learning using cFos expression as a marker of neurons undergoing learning-induced synaptic plasticity^26^. We compared the number of cFos^+^ cells in the OBS at the end of the SDM observation phase (Fig. 4a). We found cFos^+^ cells in several brain regions; however, notably, the OBS had a higher proportion of cFos^+^ cells in the dCA1 region than their DEM (Fig. 4a) and their other brain regions.

We investigated dendritic spine density and morphology to explore structural modifications possibly associated with observational learning in dCA1. Structural plasticity changes have been observed in various learning paradigms and learning shapes neural circuits by modifying synapses^27^. We morphologically analyzed the dCA1 neurons in the DEM and OBS 6 h after the last session (day 5) of the observation phase. As a control, we used OBS separated from their DEM by an opaque partition (Fig. 4b). We found dCA1 spine density to differ between the two experimental conditions (metal mesh, opaque partition). The DEM and OBS tested with the metal mesh had a higher spine density than those tested with the opaque partition (Fig. 4c). We did not detect any difference in spine length and head width (Fig. 4c). Furthermore, we found an increased percentage of mushroom spines in the dCA1 region of OBS tested with the metal mesh than in their DEM and OBS tested with the opaque partition (Fig. 4d). These results indicate that observation induced neuronal structural changes in the dCA1.

We then decided to assess which DEM behavior could potentially influence the OBS learning process and activate dCA1 neurons in the OBS during the observation phase. To identify neurons activated in the dCA1 across sessions during the observation of the SDM we injected a Cre-dependent adeno-associated virus (AAV) carrying the fluorescent marker mCherry into Fos^2A-iCreER^ mice (or ‘TRAP2’). Administering 4-hydroxytamoxifen (4-OHT) immediately before the observation generated dCA1-activated neurons in the OBS (Fig. 4e) and we correlated the number of activated neurons (mCherry^+^) with DEM behavior. We found that the number of dCA1 mCherry-expressing neurons correlated positively with the number of DEM nose pokes associated with prosocial choices (Fig. 4f), which could reflect the encoding of the shared reward and/or reward-location association. We did not find correlations with other measurements, such as food delivery to DEM only (Fig. 4g).

### dCA1 is required for observational learning of reward and social outcome association

**Fig. 5:**
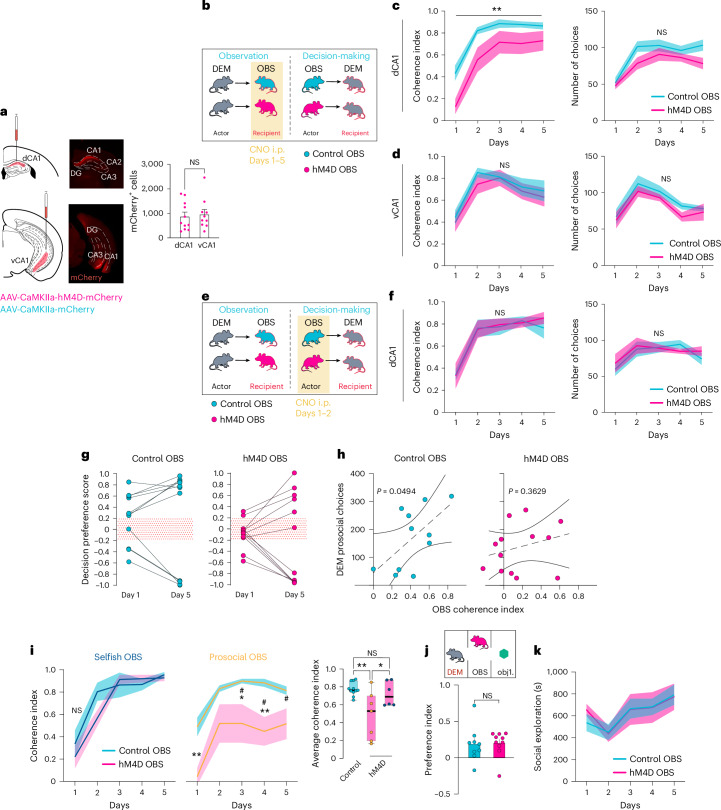
dCA1 is required for observational learning of reward and social outcome association. **a**, Inhibitory DREADDs receptors (AAV-CaMKIIa-hM4D-mCherry) or control (AAV-CaMKIIa-mCherry) were expressed in the dCA1 and vCA1 of OBS mice. Number of transduced cells in dCA1 and vCA1 (two-tailed, unpaired *t*-test, *t* = 0.328, d.f. = 19, *P* = 0.7461). **b**, Control and CA1-silenced mice received i.p. CNO injection before daily session of the observation phase of the SDM, and then were tested in the decision-making phase without CNO. **c**, Coherence index (left, two-way RM ANOVA, group, *F*_(1,21)_ = 7.46, *P* = 0.0125; control *n* = 11, hM4D *n* = 13) and number of choices (right, time (days) × group (hM4D, control), *F*_(4,84)_ = 1.26, *P* = 0.2903, hM4D *n* = 13; control *n* = 11) of dCA1-silenced and control OBS during observation. **d**, Coherence index (left, *F*_(1,16)_ = 0.53, *P* = 0.4756; control *n* = 9, hM4D *n* = 10) and number of choices (right, *F*_(4,64)_ = 9.94, *P* = 0.9603, hM4D *n* = 10; control *n* = 8) of vCA1-silenced OBS and control OBS during observation. **e**, Control and dCA1-silenced OBS received i.p. CNO injection before day 1 and 2 of the decision-making phase of the SDM. **f**, Coherence index (left, time (days) × group (hM4D, control), *F*_(4,48)_ = 0.27, *P* = 0.8934, hM4D *n* = 7; control *n* = 7) and number of choices (right, *F*_(4,48)_ = 0.60, *P* = 0.6616). **g**, Decision preference scores of control (*n* = 11) and hM4D dCA1 (*n* = 13) OBS. **h**, Correlation between coherence index of control (*n* = 11) and hM4D dCA1 (*n* = 13) OBS on day 1 of the decision-making phase of the SDM and number of their DEM prosocial choices (linear regression). **i**, Coherence index of control OBS and hM4D dCA1 OBS grouped by prosocial (control *n* = 7, hM4D dCA1 *n* = 6) and selfish (control *n* = 5, hM4D dCA1 *n* = 6) preference (two-way RM ANOVA, group, *F*_(3,19)_ = 8.62, *P* = 0.0008, time, *F*_(4,76)_ = 28.79, *P* < 0.0001). Inset: average coherence index during the decision-making phase of the SDM of control and hM4D mice grouped by prosocial and selfish preference (one-way ANOVA, *F*_(2,20)_ = 5.85, *P* = 0.0099). **j**, Preference index of OBS mice tested in the three-chamber test with their DEM and with an inanimate object (two-tailed unpaired *t*-test, *t* = 0.1257, d.f. = 17, *P* = 0.9014). **k**, Social exploration on control and hM4D OBS toward their DEM during the 5 days of observation phase of the SDM (two-way RM ANOVA, time (days) × group (hM4D, control), *F*_(4,75)_ = 0.17, *P* = 0.9496, hM4D *n* = 9; control *n* = 8). ^#^*P* < 0.05 versus hM4D selfish, *P < 0.05, **P < 0.01 versus control. Error bars and shading represent the s.e.m. unless otherwise noted. *P < 0.05; **P < 0.01; ***P < 0.001; NS, not significant.

To determine whether the dCA1 has a causal role in learning the socially relevant contingencies of DEM actions, we used inhibitory chemogenetics during the observation phase of the SDM (Fig. 5a,b). As a control, we injected mice in the ventral hippocampal CA1 (vCA1). Mice received an AAV carrying the inhibitory designer receptor exclusively activated by designer drugs (DREADD) receptor hM4D or a control virus, under CaMKIIa promoter, into the dCA1 or the vCA1 (Fig. 5a). Both hM4D and control OBS mice were injected daily with clozapine-*N*-oxide (CNO) 30 min before each session to silence the dCA1 or vCA1 during the entire observation phase of the SDM (Fig. 5b). At the end of DEM performance, hM4Di and control OBS were then tested as decision-makers in the SDM.

Chronic neuronal silencing in the dCA1 but not the vCA1 impaired observational learning (Fig. 5c,d), as the coherence index was lower in hM4D dCA1 OBS than in control OBS (Fig. 5c). In contrast, hM4D dCA1 OBS did not display any difference in the number of choices (Fig. 5c), indicating that dCA1 silencing did not affect the nose poke–outcome association. Next, to understand the specific contribution of the dCA1, we tested an additional group of OBS that received inhibitory DREADDs in the dCA1 (hM4D dCA1) or mCherry (control) and were injected with CNO for silencing only during the decision-making phase (day 1 and 2). We found no differences in the coherence index and number of choices between hM4D dCA1 and control OBS (Fig. 5f). Therefore, these results demonstrate that dCA1 is required for the acquisition of socially mediated action–outcome association, but not for later recall of social memories.

To gain greater insight into the role of the dCA1 in this behavior, we checked the social preference of OBS after chemogenetic silencing. The OBS in the control group displayed a clear preference, either prosocial or selfish, on day 1 (10 of 11; Fig. 5g). Instead, most hM4D dCA1 OBS (8 of 12) did not show a clear social preference (Fig. 5g), and they displayed coherent choices only in later daily sessions suggesting that dCA1 silencing did not affect the learning of procedural aspects but rather affected the association between DEM choices and their consequences. Further, we found that dCA1 silencing abolished the positive correlation between the coherence index of OBS and the number of prosocial choices made by their DEM during the observation phase (Fig. 5h). These results indicate that the dCA1 might be necessary for the OBS to integrate information about the DEM choices during observation.

Then, we analyzed the hM4D dCA1-silenced OBS based on their displayed preference in the decision-making phase. We found that only OBS displaying a preference for prosocial choices showed lower coherence scores than the control OBS (Fig. 5i). We did not find differences in other motivational behaviors such as sociability (Fig. 5j) and social exploration (Fig. 5k) in the dCA1-silenced mice. These results suggest that dCA1 silencing might have affected learning, particularly of those with a propensity for prosocial choices.

### Observational learning recruits distinct dCA1 neuronal patterns

**Fig. 6:**
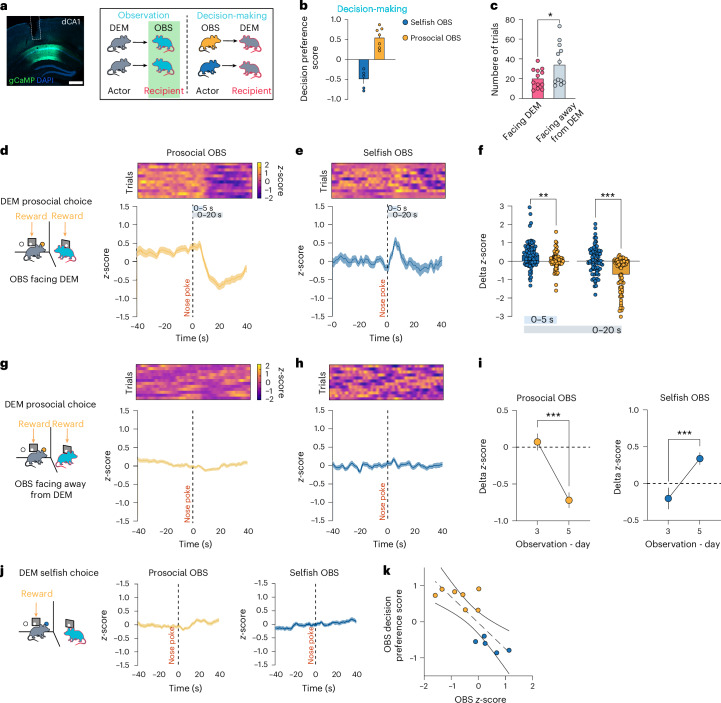
dCA1 neuronal patterns of observed prosocial choices. **a**,**b**, Expression of GCaMP6f in the dCA1 (**a**, left). Scale bar 500 μm. OBS were injected with a virus encoding GCaMP6f (AAV-CaMKIIa-GCaMP6f) in the dCA1 and recorded during the observation phase of the SDM (**a**, right). Recordings were analyzed separately for OBS displaying prosocial (*n* = 7 mice) or selfish (*n* = 5 mice) preference in the decision-making phase (**b**). **c**, Number of trials in which OBS faced or faced away from DEM (two-tailed unpaired *t*-test, *t* = 2.099, d.f. = 22, *P* = 0.0475, *n* = 12 mice). **d**,**e**, Representation of GCaMP6f fluorescence changes in the dCA1 of prosocial OBS (**d**) and selfish OBS (**e**) time-locked to prosocial choices of DEM day 5 of observation when facing DEM during nose poking. **f**, Quantification of GCaMP6f fluorescence changes within 5 s (left, two-tailed unpaired *t*-test, *t* = 3.098, d.f. = 159, *P* = 0.0023, prosocial OBS *n* = 81 trials from seven mice, selfish OBS *n* = 80 trials from five mice) and 20 s (right, *t* = 5.534, d.f. = 148, *P* < 0.0001, prosocial OBS *n* = 75 trials from seven mice, selfish OBS *n* = 75 trials from five mice) following DEM prosocial choice. **g**,**h**, Representation of GCaMP6f fluorescence changes in the dCA1 of prosocial OBS (**g**, *n* = 130 trials from seven mice) and selfish OBS (**h**, *n* = 134 trials from five mice) time-locked to prosocial choices of DEM day 5 of observation when facing away from DEM during nose poking. **i**, Quantification of GCaMP6f fluorescence changes upon DEM prosocial choice between day 3 and day 5 (day 3 *n* = 27, day 5 *n* = 75 trials) in prosocial OBS (left, *t* = 4.210, d.f. = 101, *P* < 0.0001, from *n* = 7 mice) and selfish OBS (right, *t* = 3.488, d.f. = 117, *P* = 0.0007, day 3 *n* = 39, day 5 *n* = 80 trials, from *n* = 5 mice). **j**, GCaMP6f fluorescence changes in the dCA1 of prosocial (left*, n* = 125 trials from seven mice) and selfish OBS (right, pokes OBS, *n* = 83 trials from five mice) in response to DEM selfish nose. **k**, Correlation between OBS decision preference score (orange, prosocial, and blue, selfish) and OBS mean *z*-score (0–20 s) following DEM prosocial choices (simple linear regression, *r*^2^ = 0.6371, *P* = 0.0019*, n* = 12 mice). Error bars and shading represent the s.e.m. unless otherwise noted.

**Extended Data Fig. 6:**
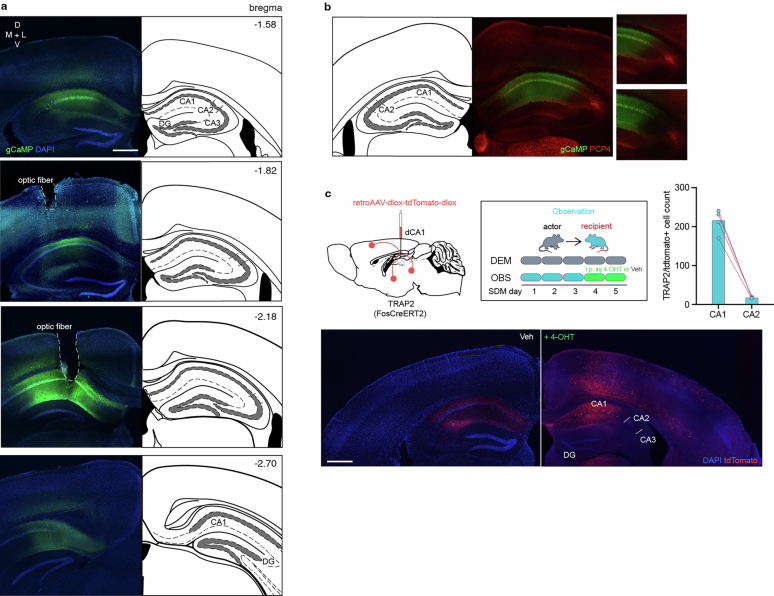
Validation of dCA1 targeting and CA2 engagement during observational learning. **a**, *Left*, Rostro-caudal reconstruction of GCaMP expression (and location of optical fibers) in OBS mice injected with AAV1-CamKII.GCaMP6f.WPRE.SV40 in the dCA1, and, *right*, adapted reference atlas images^50^ with distance from bregma. Scale bars (applicable to all micrographs), 500 μm. **b**, Merge of PCP4 and GCaMP staining (red, *n* = 3 mice) showing no overlap. **c**, OBS TRAP2 mice were infused with AAV-retro/2-hSyn1-dlox-tdTomato(rev)-dlox-WPRE-bGHp(A) in the dCA1 and injected with 4-OHT (or Veh in control OBS mice) to label active population of neurons during observation (days 4-5). Quantification of tdTomato-positive-TRAPed cells in the dCA1 and dCA2 of OBS mice. *Bottom*, representative images of OBS mice (*n* = 4) injected with 4-OHT or Veh on day 4-5 of observation. Scale bar 500 μm.

To understand the functional role of the dCA1 when observing DEM selfish and prosocial choices, we used fiber photometry to directly monitor neural activity in the dCA1 of OBS during the observation phase of the SDM (Fig. 6a and Extended Data Fig. 6a). We injected a virus carrying a genetically encoded fluorescent calcium indicator (GCaMP6f), under CaMKIIa promoter, into the dCA1 of the OBS. Then, the OBS were tested in the decision-making phase to assess their preference, and the recordings were analyzed based on their later preference for prosocial or selfish choices (Fig. 6b). Using SimBA^23^ we quantified the number of trials in which the OBS were facing the DEM based on their body position and could witness their nose poking. Because we found a higher number of events wherein OBS were facing away from DEM (Fig. 6c), we analyzed the recordings based on the likelihood of the OBS witnessing their DEM’s actions.

We found a suppressed and prolonged (≅40 s) neural activity in OBS time-locked to the DEM prosocial choices (Fig. 6d,f) on the last day of observation (day 5). This effect was not present on day 3 (Fig. 6i), indicating that suppression increased when the task was likely acquired. We did not detect a similar neuronal suppression in the selfish OBS (Fig. 6e,f). In contrast, the selfish OBS show a rapid, transient (≈5 s) increase in neuronal activity upon DEM prosocial choices (Fig. 6e), which was not detected on day 3 (Fig. 6i).

Furthermore, the neuronal patterns we identified in prosocial and selfish OBS on day 5 were not present when the OBS were not facing DEM upon nose poking for food sharing (Fig. 6g,h). Because OBS received a food reward both when facing or facing away from DEM, these results suggest that dCA1 neuronal activity does not depend on reward delivery or consumption per se, but rather it is related to the prediction or the value of the reward, associated with the DEM action, or to differential behavioral responses of the OBS following shared reward. These results are in line with the above findings showing that dCA1 activity correlates with number of shared rewards (Fig. 4f). Furthermore, these neuronal patterns in prosocial and selfish OBS were evident only upon DEM prosocial but not selfish choices (Fig. 6j). Notably, differential levels of dCA1 neural activity correlated positively with OBS decision preference scores (Fig. 6k).

Previous studies have found that dorsal CA2, and not CA1, is important for socially relevant forms of memory^28,29^. Thus, to investigate the specificity of CA1 expression of the calcium indicator in the neural recordings we co-stained for a known CA2 pyramidal neurons marker, which showed no overlap with GCaMP expression (Extended Data Fig. 6b). Hippocampal CA2 also present dense projections to CA1^30,31^. Thus, to assess the involvement of CA2 during observation of social behaviors we injected OBS Fos^2A-iCreER^ mice with a retro-AAV-hSyn1-dlox-tdTomato-dlox to label active population of cells connected to the CA1 (Extended Data Fig. 6c). In addition to activation within the CA1 local network, we also detected activated cells in the CA2 (Extended Data Fig. 6c)^29,30^. All these results suggest that task learning during observation manifests as distinct dCA1 neural signatures that correspond to different behavioral outcomes in the decision-making.

**Fig. 7:**
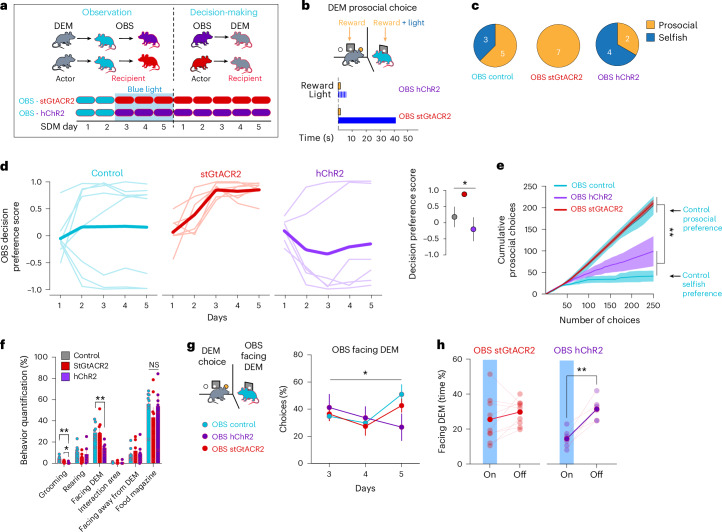
Optogenetic manipulation of dCA1 activity during observation causally shapes prosocial behavior. **a**, Optogenetic activation (hChR2) or inhibition (stGtACR2) in the bilateral dCA1 of OBS during observation of prosocial choices from day 3 to 5. OBS were then tested for 5 days in the SDM without manipulations. **b**, Following DEM prosocial choice and concurrent reward delivery OBS received 5 s of photo stimulation (hChR2) or 40 s of photo inhibition (stGtACR2). **c**,**d**, Number of OBS (**c**, chi-squared test, 6.653, d.f. = 2, *P* = 0.0376) showing preference for prosocial and selfish choices, and decision preference scores of control (*n* = 8), stGtACR2 (*n* = 7) and hChR2 (*n* = 6) OBS (**d**) during the 5 days of the decision-making phase of the SDM (two-way RM ANOVA, group × time, *F*_(4,76)_ = 28.79, *P* = 0.0012; inset: average decision preference of the last 3 days of the decision-making phase, one-way ANOVA, *F*_(2,18)_ = 3.73, *P* = 0.0440). **e**, Cumulative number of prosocial choices (two-way RM ANOVA, group × time, *F*_(747,4233)_ = 4.07, *P* < 0.0001) in hChR2 (*n* = 6), and stGtACR2 (*n* = 7) OBS compared to control OBS (*n* = 8) grouped by preference (prosocial, selfish) during the 5 days of the decision-making phase of the SDM. **f**, Quantification of control, stGtACR2 and hChR2 OBS behaviors during the observation phase of the SDM (two-way RM ANOVA, group (control, hChR2 and stGtACR2), *F*_(2,16)_ = 10.03, *P* = 0.0015). **g**, Number of DEM prosocial choices (in %) witnessed by OBS (control OBS *n* = 8, OBS hChR2 *n* = 6, OBS stGtACR2 *n* = 7) over 3 days of optogenetic manipulations (two-way RM ANOVA, group × time, *F*_(4,36)_ = 2.76, *P* = 0.0420). **h**, Time spent (in %) facing DEM during ‘light on’ and ‘light off’ periods in and stGtACR2 (two-tailed paired *t*-test, *t* = 1.168, d.f. = 8, *P* = 0.2763, *n* = 9) and hChR2 OBS mice (*t* = 5.274, d.f. = 5, *P* = 0.0033 *n* = 6). Data are shown as mean ± s.e.m. *P < 0.05; **P < 0.01; NS, not significant.

To directly determine whether the different neuronal patterns in prosocial and selfish OBS play a causal role in observational learning, we first used reversible optogenetic inhibition of dCA1 neurons in the OBS specifically only following DEM prosocial choices, during the last 3 days of observation (Fig. 7a). The dCA1 pyramidal neurons of the OBS were injected with an inhibitory opsin (stGtACR2). Guided by the fiber photometry experiment, we set the photo inhibition for 40 s after DEM prosocial choices (Fig. 7b) and found that all stGtACR2 OBS displayed a preference for prosocial choices (Fig. 7c).

To further explore whether dCA1 neuronal dynamics underlying observation of prosocial actions play a causal role in biasing subsequent choices, we injected an excitatory opsin into the dCA1 (hChR2). The OBS received optogenetic activation of the dCA1 neurons upon DEM prosocial choices (Fig. 7b). Most hChR2 OBS displayed a preference for selfish choices (Fig. 7c) and, at the group level, showed a negative decision preference score (Fig. 7d). Indeed, the hChR2 OBS made fewer prosocial choices compared to the stGtACR2 OBS (Fig. 7e). Thus, these results show that specific inhibition of dCA1 activity (stGtACR2 OBS) after DEM prosocial choices promoted prosocial behavior in the OBS, while dCA1 activation (hChR2 OBS) following these events produced opposite effects and biased the OBS toward selfish choices (Fig. 7d). Furthermore, we quantified other OBS behaviors during the optogenetic manipulation, and we found that hChR2 OBS showed reduced time spent facing their DEM (Fig. 7f), also during DEM choices, resulting in reduced number of witnessed DEM nose poking (Fig. 7g); however, time facing the DEM could be reestablished during light off periods in hChR2 OBS (Fig. 7h). Together, these results indicate that distinct dCA1 activity patterns during observation of prosocial actions causally shape a bias for prosocial or selfish choices in future social interactions.

## Discussion

Our study showed that mice learn the association between an action and its social consequences through socially transmitted information, similar to what happens in observational threat learning. We show that this behavior is goal-directed and flexible. We investigated the neurobiology of observational learning by establishing a procedure in which mice learn to share food rewards by observing a conspecific. We found that the dCA1 is required for observational learning; it induced plasticity-dependent changes and engaged neuronal activity that differ according to mice behavior in future social interactions.

The variability in observational learning was influenced by the DEM’s willingness to share rewards, with OBS learning positively correlating with DEM prosocial behavior; however, OBS did not simply mirror their model, suggesting a role for individual propensity. In this sense, our study revealed that OBS mice exhibit a general tendency to make more prosocial choices than their DEM, even when they had primarily witnessed selfish DEM, and irrespective of their hierarchical (subordinate) status. This challenges previous work suggesting dominance predicts prosociality^18^ and indicates that even infrequent prosocial actions provide sufficient information for behavioral plasticity, potentially representing an adaptive mechanism that promotes social cohesion regardless of hierarchical position. Of note, OBS choice pattern is not strictly determined by the DEM behavior. Instead, our results suggest that two interrelated mechanisms may be at play: (1) repeated vicarious experience of reward could strengthen the association between DEM action and its positive outcome for the OBS, reinforcing the value of the prosocial option through associative learning mechanisms; and (2) the social context in which rewards are shared may generally increase the motivating appeal of prosocial options, thereby leading OBS to favor these choices when they subsequently make decisions themselves. In both scenarios, the social context enhances the salience of prosocial outcomes, supporting faster and more robust learning. Nevertheless, the role of individual variability and context-dependent learning processes in the shaping of observed behavior remains a complex interplay to dissect. In addition to variability linked to DEM behavior, we did not observe sex differences in observational learning, despite previous reports in direct reinforcement setting^18^. This apparent discrepancy may reflect differences in how social information is processed or utilized by each sex. Observational learning could provide additional social cues or contextual information that enable female mice to overcome more conservative decision-making tendencies.

What neural mechanism underlies the differences between mice that engage in prosocial behavior and others that do not? Our study found distinct patterns of dCA1 neuronal activity during observation of social behaviors that correlated with preferences in future social interactions. The number of prosocial choices made by the DEM correlated positively with the levels of dCA1 neuronal activation and plasticity changes. These results are consistent with a recent study showing that the dorsal hippocampus regulate helping behavior^32^, a form of prosocial, empathy-driven, behavior, in mice. This convergence of evidence suggests that the hippocampus may serve as an interface between cognitive processing and emotional networks underlying prosocial decision-making. Accumulating evidence shows how the hippocampus is required also to recall socially acquired information related to aversive stimulus^8,33^, which, in turn, supports survival through danger avoidance^34^. In our study, dCA1 inactivation impaired the acquisition but not the later recall of information during decision-making. Furthermore, our findings point to a crucial role for the dCA1, but not dCA2 and vCA1 in the acquisition of social behaviors, which indicates the involvement of a neural pathway that could be distinct from the one activated for social memory^29,35^; however, our data do not exclude the potential involvement of CA2 or ventral areas of the hippocampus, which may be more engaged during earlier observation stages when social recognition memory is critical, rather than in later phases when the task has been largely acquired.

Prosocial tendencies observed across diverse animal taxa points to an adaptive basis for such behaviors^36^. Experimental evidence demonstrates that some mammals, including rodents, display spontaneous prosocial behaviors even in the absence of previous specific training^14,15,37^. In this light, our finding that chemogenetic silencing of dCA1 during observational learning selectively impaired the acquisition in individuals displaying preference for prosocial choices (and not those displaying preference for selfish) may reflect individual variability that may take into account other factors that shape the predisposition for flexible expressions of prosociality. Our findings align with recent work^38^ demonstrating that dCA1 neurons selectively represent reward expectation in social contexts and maintain dynamic representations of social identities. Together, these studies suggest that the dorsal hippocampus could contain neurons for processing socially relevant information and rewards, which may be particularly crucial for acquisition of prosocial actions, also through observational learning.

Our study extends previous results showing that, among social and nonsocial factors^17,18,25^, neurobiological determinants could define how prosocial behaviors are acquired and subsequently displayed. A subpopulation of BLA neurons projecting into the prefrontal cortex (PFC) is involved in encoding the social values of rewards for self and others^18^. Therefore, a shared circuit, encompassing the PFC, the BLA and the hippocampus, could represent the neural basis for learning social behaviors, either by self-experience or observation. The differential engagement of this neural circuitry may thus represent a key substrate through which social learning can guide future social interactions.

While chemogenetic prolonged inhibition established dCA1 role in the observation phase, targeted optogenetic manipulations identified critical temporal windows for dCA1 contribution. Neuronal recordings revealed complex patterns where prosocial OBS showed reduced dCA1 activity specifically following witnessing prosocial choices on day 5, but not day 3. This selective reduction in activity does not preclude broader dCA1 engagement during observational learning, as evidenced by increased cFos expression and enhanced synaptic plasticity. These complementary approaches reveal that dCA1 activity during observation is necessary for encoding socially structured contingencies, and that its temporal dynamics shape subsequent choice biases.

Similar region-specific hippocampal activity has been reported in goal-directed behaviors^39^. Dorsal and ventral hippocampal regions exhibited distinct activity patterns during operant training with dCA1 activity declined, whereas vCA1 activity progressively following reward delivery. Although with differences in latencies, related to longer delays retrieving food rewards, in our study, we observed sustained dCA1 suppression in prosocial OBS following witnessed prosocial choices, contrasting with the transient activation found in selfish OBS, suggesting potential differential engagement of hippocampal circuitry based on social preference. These findings align with human neuroimaging data showing that, using a dictator game, socially adaptive value-based choices were associated with different degrees of hippocampus activity suppression^40^. Together, we showed complementary roles of hippocampal subregions in goal-directed behaviors, with dCA1 supporting socially transmitted information acquisition and vCA1 enabling subsequent execution of learned behaviors.

The OBS displayed behavioral flexibility rather than simple imitation, adapting their behavior when task features changed (relocation of nose poke holes) and generalizing it across conditions, including partner identity (familiar versus unfamiliar) and presence versus absence of a recipient. The lack of side-specific copying indicates that OBS did not merely reproduce the DEM’s motor pattern but instead learned that certain actions have consequences for a social partner. This learning can be explained within standard associative mechanisms. Our findings suggest that the dCA1 allows flexible adaptation of the observed behavior to guide future choices under novel circumstances. These results extend previous findings on rodent observational learning, demonstrating the flexibility and adaptability of this behavior to changing environmental conditions^41^. In this frame, our findings add a new facet supporting a role for the dCA1 in forming a cognitive map^42^, which is not limited to physical space but involves other spaces, such as the social one^43^.

Overall, our study characterized the role of the dCA1 in learning action–outcome contingency in a social context using a different perspective: the point of view of the OBS or partner. Our results have noteworthy implications for studying neuropsychiatric, psychopathological and neurodevelopmental conditions that are often associated with social learning impairments that could limit the capacity to form and sustain interpersonal relationships in our society.

## Methods

### Mice

All procedures were approved by the Italian Ministry of Health (permit nos. 191/2020-PR and 200/22-PR) and local Animal Use Committee and were conducted in accordance with the Guide for the Care and Use of Laboratory Animals of the National Institutes of Health and the European Community Council Directives. Routine veterinary care and animals’ maintenance was provided by dedicated and trained personnel. We used 3–6-month-old male and female C57BL/6J animals. For targeted recombination in active populations (TRAP) experiments, we used 2–6-month-old male and female second-generation of fos-TRAP mice (Fos2A-iCreER;TRAP2, RRID:IMSR_JAX:030323). Distinct cohorts of naive mice were used for each experiment. Animals were housed two to four per cage in a climate-controlled facility (22 ± 2 °C), with ad libitum access to food and water throughout, and with a 12-h light–dark cycle (19:00–7:00 schedule). Experiments were run during the light phase (within 10:00–17:00). All mice were handled on alternate days during the week preceding the first behavioral testing.

### Behavioral paradigms

#### Social decision-making task

##### Experimental setup

Experiments were conducted in a standard operant chamber (the actor/DEM compartment, length 24 cm × width 20 cm × height 18.5 cm; ENV-307W-CT; Med Associates) fused with a custom-made small triangle-shaped chamber (the recipient/OBS compartment, length 18 cm × width 14 cm × height 18.5 cm). The separation wall between the two compartments consisted of a metal mesh with 1 cm holes that allowed social exploration and nose-to-nose interaction. The DEM compartment was equipped with two nose poke holes and a food magazine between them, for delivery of food rewards (14 mg; Test Diet, 5-TUL). The OBS compartment presented only a food magazine connected to a food dispenser. In the opaque partition condition, the metal mesh was replaced with an opaque plastic partition, and in the one-way mirror condition with a transparent plastic partition with adhesive one-way mirror film of the same size dividing DEM and OBS during the SDM observation phase. The setup was placed inside a sound attenuating cubicle (ENV-022V, Med Associates) homogeneously and dimly lit (6 ± 1 lux) to minimize gradients in light, temperature, sound and other environmental conditions that could produce a side preference. All the setup was controlled by custom scripts written in MED-PC IV (Med Associates). Furthermore, a digital camera (Imaging Source, DMK 22AUC03 monochrome) was placed on top of the setup to record the test using a behavioral tracking system (Anymaze 7.3, Stoelting).

##### Task design

The task was adapted from a previous study. Each cage, containing both DEM and OBS, hosted two to four mice for at least 2 weeks before the experiment. All DEM–OBS pairs were composed by cage mates. Animals were mildly food-restricted to 90–95% of their baseline weight to encourage task engagement in the initial phase and. During the SDM observation phase, DEM could determine whether to receive a food reward for themselves (the selfish choice), or to allocate the reward also to the OBS (the prosocial choice) (Fig. 1a). Both choices were reinforced on a fixed ratio of 1, such that one nose poke either to the left or the right side corresponded to one food reward delivery. After one nose poke, an intertrial interval of 5 s occurred, in which both nose poke holes were inactive, and DEM could retrieve a food pellet. During the SDM observation phase, OBS were passive players, only receiving food rewards upon DEM choices. In this phase, DEM were tested for 5 days in 40-min sessions and were always paired with the same OBS throughout the experiment. During the SDM decision-making phase, OBS were placed in the DEM compartment and tested using the same two-choice scheme previously used for the DEM for 5 days in 40-min sessions. Nose poke holes associated with prosocial and selfish choices were counterbalanced across DEM and OBS and were never changed across test sessions for the same mouse. The location of the prosocial choice (nose poke configuration) was maintained the same across the 10 days of testing (observation and decision-making phase) for each DEM–OBS dyad. To test OBS with a different nose poke configuration the location of the prosocial choice was moved to the opposite side during the decision-making phase to test OBS flexibility.

##### Analyses

The number of nose poke responses was counted by the MED-PC software (MED-PC V, Med Associates) and then imported by using the MED-PC to Excel tool (Med Associates) into a Microsoft Excel spreadsheet. To quantify individual preference for prosocial over selfish choices, we calculated a decision preference score, as (number of prosocial choices − number of selfish choices)/total number of choices. To measure learning in DEM and OBS we used a coherence index, calculated as (number of responses on preferred nose poke − number of responses on the other nose poke)/total number of nose pokes responses. The coherence index provides a quantification of coherence of the social preference displayed in subsequent sessions throughout the task. Video images were analyzed a posteriori for scoring exploratory behavior using Anymaze 7.3 (Stoelting) and Boris 8.22.14 (University of Turin)^44^. By using Anymaze, we measured the time spent by OBS in social exploration in an area proximal to the compartment where the DEM were present.

##### Automatic markerless pose estimation and behavioral classification

To build the DeepLabCut (DLC) model, 1,517 frames were extracted from 33 videos and labeled using the graphical user interface (GUI), providing animals’ bodyparts (we tracked six points per animal, consisting of nose, left and right ears, center, tail base and tail end) and identities (DEM and OBS). Frame extraction was conducted through *k*-means or uniform method. A multi-animal dataset containing 95% of total frames was created to train the model. To evaluate and test the dataset, the remaining 5% of the total frames were used. The DLC model was locally trained with an NVIDIA GPU GeForce GTX 1650 Ti. The multi-animal model/network (dlcrnet_ms5) was trained for 100,000 iterations as suggested by Lauer et al.^22^. The pose estimation data were imported to SimBA for further analysis and video files (mp4) with animals’ identities for visually evaluating the model’s efficiency. In the case of identity swaps, videos were manually refined in the DLC GUI (refine tracklets), predictions were filtered and further used for generating the necessary CSV files.

After extracting tracking data (CSV files) from the DLC, videos containing OBS and DEM were analyzed for further behavioral classification using SimBA. We used SimBA region of interest (ROI) and directionality tools^23^ to make a proxy calculation for estimating whether the OBS was directed toward the DEM or toward user-defined ROIs in the DEM compartment. To do this SimBA required pose estimation tracking of the two ears and the nose of each animal, and the center coordinate of the ROIs. Specific ROIs drawn in the DEM compartment included the ‘interaction area’, DEM nose poke holes and DEM food magazine. Furthermore, SimBA estimated the time OBS spent directed toward DEM and the time DEM and OBS spent directed toward each other (Fig. 1j).

#### Three-chamber task

For assessing sociability in control and dCA1-silenced OBS mice were tested in a standard three-chamber sociability cage (Ugo Basile, 60 × 40 × 22cm) equipped with transparent PVC walls and two cups that could host either a mouse or an object (15 cm in height, diameter 7 cm). After each test, the apparatus was cleaned with 50% ethanol and allowed to air dry. All test stages were carried under dimly lit (6 ± 1 lux). A digital camera (Imaging Source, DMK 22AUC03 monochrome) was placed above the apparatus to record the test using a behavioral tracking system (Anymaze 7.3, Stoelting). Habituation (10 min) to the apparatus with empty cups occurred on the test day. After the habituation, the sociability/social preference phase (10 min) started, and the OBS had the possibility to investigate both sides of the chamber where either an unfamiliar object, built with Duplo blocks (Lego), or an unfamiliar mouse were placed inside the cups.

For testing OBS preferences in a different experimental setting following the SDM the original partner (DEM) of the OBS was placed in one of the cups at one side of the apparatus, whereas the other cup contained an object. The day after, as a control, a novel unfamiliar partner, was placed into one of the cups, while the other contained a new object. Here, OBS were tested for a further 10 min to assess the preference for the new partner over the object. At the end of the procedure, mice were returned to their home cage.

#### Social interaction task

The social interaction task was conducted in a standard rat cage (Tecniplast, 425 × 266 × 185 mm, floor area, 800 cm^2^). First, mice were singularly habituated to the arena (identical to the test arena) for 30 min and then were simultaneously placed in the test arena for 10 min free interaction. The test was performed inside a sound-proof cubicle (Ugo Basile fear-conditioning cubicle, outer size of 52 × 62 × 58cm, internal size of 39 × 47 × 49cm) in dim light conditions (15 lux). The test was recorded using a digital camera (Imaging Source, DMK 22AUC03 monochrome) and then manually scored using the software Boris (v.8.22.14, University of Turin). At the end of the procedure, mice were returned to their home cage.

#### Tube test

We assessed social hierarchy before the SDM test using a transparent Plexiglas tube test (30 cm length, 3 cm internal diameter). For habituation, the tube was placed in the home cage for 3 consecutive days, followed by 2 days of training, where each mouse completed ten trials of traversing the tube, with gentle guidance when necessary. During testing, two mice were simultaneously released from opposite ends of the tube. The first mouse to retreat by placing both rear paws outside the tube was designated the ‘loser’ while the other was recorded as the ‘winner.’ The tube was cleaned with 50% ethanol between trials. Mice were tested in a round-robin tournament format, with each pair of cage mates tested in consecutive trials, while alternating their starting sides. Testing continued daily until rank stability was achieved for at least 4 consecutive days.

### Stereotaxic surgeries

#### Viral vectors

AAV5-CamKIIa-mCherry (114469-AAV5, 100 µl at titer ≥7 × 10^12^ vg ml^−1^), AAV5-CamKIIa-hM4D(Gi)-mCherry (50477-AAV5, 100 µl at titer ≥3 × 10^12^ vg ml^−1^), AAV5-hSyn-DIO-mCherry (50459-AAV5, 100 µl at titer ≥7 × 10^12^ vg ml^−1^) and AAV1-CamKII.GcaMP6f.WPRE.SV40 (100834-AAV1, 100 µl at titer ≥1 × 10^13^ vg ml^−1^) and AAV1-CamKIIa-stGtACR2-FusionRed (105669-AAV1, 100 µl at titer ≥1 × 10^13^ vg ml^−1^) were purchased from Addgene. AAV1-CaMKIIa-hChR2(H134R)-mCherry (v204-1 titer ≥6.5 × 10^12^ vg ml^−1^) and AAV-retro/2-hSyn1-dlox-tdTomato(rev)-dlox-WPRE-bGHp(A) (v284-retro titer ≥8.5 × 10^12^ vg ml^−1^) were purchased from the Viral Vector Facility of the Neuroscience Center Zurich.

#### Surgical procedures

C57BL/6J mice were naive and 2 months old at the time of surgery. All mice were anesthetized with a mix of isoflurane/oxygen 2%/1.5% by inhalation and mounted onto a stereotaxic apparatus (Stoelting) linked to a digital micromanipulator. Brain coordinates of viral injection were chosen following the mouse brain atlas^45^: dCA1, anteroposterior (AP): −2mm, mediolateral (ML): ±1.5 mm, dorsoventral (DV): −1.3 mm; vCA1, AP: -3.16 mm, ML: ±3.10 mm, DV: −4.30 mm. The volume of AAV injection was 400 nl (injection rate 0.2 μl min^−1^) per hemisphere. We infused the virus through a 10-μl Hamilton syringe. After infusion, the pipette was kept in place for 3 min (diffusion time) and slowly withdrawn. After virus injection, mice were allowed 4 weeks to recover and for the viral transgenes to adequately express before behavioral experiments.

For fiber photometry and optogenetic experiments, a 10-μl Hamilton syringe was lowered into the dCA1 (DV, −1.30 mm from the skull) and 400 nl of virus was injected (0.2 μl min^−1^) using a syringe pump (Harvard Apparatus). After infusion, the pipette was kept in place for an additional 3 min and then slowly withdrawn. A multimode fiberoptic cannula (200-μm core, 0.39 NA for fiber photometry or 0.50 NA for optogenetics, ~2 mm, RWD) was implanted 0.10 mm above the injection site (DV, −1.20 mm from the skull). The implant was secured to the skull with Super-Bond Universal kit (K058E). After surgery mice were allowed to recover for 4 weeks and for virus expression before the experiment began.

### Fiber photometry recordings

To assess the activity of dCA1 neurons during the SDM observation phase, the fluorescence signal emitted by GCaMP6f-expressing neurons was recorded using fiber photometry. Experiments were performed with a one-site, two-color Fiber Photometry System (Doric Lenses) measuring both the 405 nm isosbestic and 465 nm calcium dependent GCaMP6f fluorescence on a single photodetector. Signals were recorded at 12 kHz using the built-in lock-in mode (Doric Neuroscience Studio). In brief, 405 nm and 465 nm fiber-coupled-LEDs were sinusoidally modulated at 531 and 211 Hz, respectively, passed through an excitation filter and focused into a 400-μm fiber (NA 0.48) coupled to the mouse optic fiber implant. Emitted light was collected through the same fiber, passed through an emission filter and detected by a photoreceiver module. Animals were habituated to the patch cord once a day for 10 min for 3 days before the beginning of the behavioral experiment. Med-PC system (Med-PC V Software Suite, Med Associates) generated TTLs to time-stamp specific events (nose pokes, food receptacle entries and pellet deliveries). Acquired data files were processed with custom-written codes in Python. First, we applied the Δ*F*/*F* (signal − control)/control × 100)^46^. We used the isosbestic channel for normalization. To analyze traces around the demonstrator nose poke (PSTH analysis), we studied the deviation of the Δ*F*/*F* signal from its mean (*z*-score) extracted in epochs of ±40 s around nose pokes. Data preparation for Δ*F*/*F* involved the following stages. (1) Cutting the photometry signal by 5 s at the start and 5 s at the end. (2) Setting intervals with artifacts in the recordings to NaN. (3) Low-pass filtering the photometry traces using a zero-phase moving average forward–backward linear digital filter. The filter’s window size was 100 data points for the isosbestic control trace and for the signal trace (equivalent to 1.660 s). The filter was applied independently to each continuous section of the recordings. (4) Fitting the isosbestic control channel to the signal channel using a least-squares polynomial fit of degree 1. Subsequently, the fitted and smoothed control channel was linearly interpolated at the exact time points where the signal was sampled so that Δ*F*/*F* could be precisely computed. Finally, before PSTH analysis, we performed linear interpolation of the trace to align each epoch to the same ±40 s time points around nose poke events; epochs with artifacts (NaN values) within the ±40 s where removed.

### Optogenetic manipulations

During behavioral testing, fiberoptic cannulae were connected to patch cords (Doric Lenses), which were in turn connected to blue (447 nm) LED (Prizmatix) using a 1 x2 intensity division fiberoptic rotary joint Prizmatix) located above the cubicle containing the testing arena. LED power was adjusted such that the light exiting the fiberoptic cable was approximately 4.5 mW. For photo-inhibition experiments we used continuous blue light and LED stimulations were controlled by a microcontroller board (PulserPlus, Prizmatix). For photo-stimulation experiments we used either 5-s continuous light pulses or 30-Hz, 5-ms pulses of blue light. Animals were habituated to the patch cord once a day for 10 min inside a standard mouse cage for 3 days before the beginning of the behavioral experiment.

### Quantification of cFos-positive cells

DEM and OBS mice were tested in the SDM task for 5 days. On the last day of the SDM observation phase (day 5), mice were killed 90 min after the session and brains were collected and processed for immunohistochemical detection of cFos protein. All cells were counted bilaterally from two to four coronal sections for each brain area. Images were analyzed using Fiji–ImageJ and cFos-positive nuclei were calculated as cells per mm^2^, by dividing the number of the nuclei, automatically calculated by the software, per slide area.

### Spine morphology analysis

For confocal imaging of dendritic spines, neurons were labeled with DiI stain (1,1’-dioctadecyl-3,3,3’,3’-tetramethylindocarbocyanine perchlorate (‘DiI’; DiIC18(3); Invitrogen, D282), a fluorescent lipophilic carbocyanine dye. DiI labeling procedure was performed as previously described^47^. DiI solid crystals were applied using a thin needle by lightly touching the ROI on both sides of a 3-mm brain piece comprising the dorsal hippocampus prepared after cardiac perfusion, 6 h following the last behavioral SDM session, with 1.5% PFA in 0.1 M phosphate buffer (PB). DiI dye was left to diffuse for 1 day in the dark at room temperature, and then slices were post-fixed with 4% PFA in 0.1 M PB for 45 min at 4 °C. The first slice containing the DiI crystals were discarded and 200-μm slices were then obtained using a vibratome and collected in PB. Slices were then mounted on Superfrost glass slides (Thermo Fisher) with the histology mounting medium Fluoroshield (Sigma-Aldrich, F6182-20ML) for confocal imaging. Fluorescence images were acquired by using Zeiss Confocal LSM900 system with a sequential acquisition setting at 1,024 × 1,024 pixels resolution at 561 nm channel. *Z*-stacks of 0.45-µm steps were taken and, for each dendritic spine, length, width, and neck were manually measured using Fiji (ImageJ software). These measurements were used to classify dendritic spines into three categories (thin, stubby and mushroom). For each neuron, at least three dendrites that were at no more than 200 µm distance from the neuron soma, for a total dendritic length of about 200–400 µm, was considered. In particular, the length and the ratio between the width of head and the width of neck (Wh:Wn) were used as parameters for the classification as follows: protrusions having a length of more than 3 µm were considered as filopodia, the others as spines; spines with a Wh:Wn ratio >1.7 were considered mushrooms; spines with a Wh:Wn ratio <1.7 were divided into stubby (shorter than 1 µm) and thin (longer than 1 µm). Protrusions with a length >3 µm were qualified as filopodia and protrusions over 5 µm were excluded from the analysis. Confocal imaging was performed on a balanced number of stained neurons of the two hemispheres.

### Drugs

For hM4D activation we used intraperitoneal (i.p.) administration of CNO dihydrochloride (water soluble) (HB6149, HelloBio) dissolved in physiological saline (0.9% NaCl) at a dose of 3 mg kg^−1^ in a volume of 10 ml, 30 min before the behavioral experiments. All mice (control CNO and hM4D CNO) received i.p. CNO injections.

For labeling TRAPed neurons, 4-hydroxytamoxifen (4-OHT) (HelloBio, HB6040) was first dissolved in pure ethanol, and 50-μl aliquots of the 20 mg ml^−1^ stock were stored in the freezer (−20 °C). Then, the aliquots were further diluted in corn oil (Sigma, cat. no. s259853 and S5007) to achieve a final concentration of 10 mg ml^−1^. The i.p. injections of 4-OHT were administered on days 4 and 5, immediately before starting the SDM task at a dosage of 50 mg kg^−1^. Mice injected with 4-OHT were tested during 2h-long sessions. The longer 2-h sessions in the TRAP experiments were specifically designed to ensure optimal labeling of task-relevant neurons, as 4-OHT has a longer temporal window of action compared to standard 40-min testing sessions.

Notably, while the sessions were longer, our behavioral data show that most task engagement occurred within the first hour, similar to what we reported in our previous work^18^. Seven days after the observation phase of the SDM task, mice were killed for tissue slice preparation and immunohistochemistry.

### Tissue slice preparation and immunohistochemistry

Mice were transcardially perfused with 40 ml of 0.1 M phosphate buffered saline (PBS) and then with cold paraformaldehyde (PFA; 4% in PBS 1×). The brain was removed from the skull and post-fixed with 4% PFA in PBS for 2–6 h at 4 °C. The brain was sliced into 50-μm coronal sections using a vibratome 1000 Plus Sectioning System (3M). To examine cFos expression, brain slices were permeabilized in 0.5% Triton X-100 in PBS (0.3% T-PBS) for 30 min at room temperature (RT) while shaking. After permeabilization, brain slices were blocked with 0.5% Triton X-100 in PBS (0.1% T-PBS) supplemented with 4% normal goat serum (NGS) for 2 h at RT, while shaking. After permeabilization and blocking, slices were incubated with anti-cFos antibody in 0.5% T-PBS supplemented with 4% NGS overnight at 4 °C, while shaking. The appropriate Alexa Fluor-conjugated secondary antibody in 0.5% T-PBS with 4% NGS was applied for 2 h at RT followed by 15 min nuclei staining with the fluorescent dye DAPI (1:50,000 in PBS; Thermo Fisher Scientific).

To detect the viral expression of mCherry, stGtACR2-FusionRed, hChR2-mCherry in dCA1 neurons, brain slices were incubated in 1% Triton X-100 in PBS (1% T-PBS) supplemented with 10% NGS for 1–2 h at RT, shaking. After permeabilization and blocking, slices were incubated with anti-dsRED polyclonal antibody in 0.3% PBS (0.3% T-PBS) supplemented with 1% NGS overnight at RT or 48 h at 4 °C, shaking. The appropriate Alexa Fluor-conjugated secondary in 0.3% PBS (0.3% T-PBS) with 1% NGS was applied for 2 h at RT followed by nuclei staining with DAPI. To visualize the viral expression of hM4D and GCaMP6f and fiber placement, dCA1- and vCA1-containing brain slices were acquired with a Nanozoomer S60 (Hamamatsu), using constant settings, or an Axiovert 200M microscope (Zeiss).

### Antibodies

For immunohistochemistry analyses, the following primary antibodies were used: rabbit anti-cFos (cat. no. 2250, Cell Signaling, dilution 1:500), rabbit anti-DsRed (cat. no. 632496, Takara; dilution 1:1,000) and rabbit anti-PCP4 (cat. no. HPA005792; Sigma-Aldrich; dilution 1:200). The following secondary antibodies were used: goat anti-rabbit-Alexa488 (cat. no. A-11034, Invitrogen; dilution 1:1,000) and goat anti-mouse Alexa647 (cat. no. A-21235, Invitrogen; dilution 1:1,000).

### Statistics and reproducibility

No statistical methods were used to predetermine sample sizes, but sample size was selected based on previous experience and estimation from related studies^18^. Animals were randomly assigned to control and manipulation groups. Experimenters were not blinded during data acquisition, but all analyses were performed with blinding to the experimental conditions. Two mice were excluded from data collection because they showed little motivation to engage in nose poke responses (fewer than ten total pokes), two were excluded because viral expression patterns were not appropriate (outside the target region) and two were excluded due to fiber misplacement and loss. Statistical analyses and figure plotting were performed using Prism v.9 (GraphPad). Data are reported as mean ± s.e.m. Statistical methods used in this study include two-way RM ANOVA and one-way ANOVA followed by Bonferroni or Tukey corrections, and Wilcoxon matched-pairs signed-rank tests, as appropriate. Single-variable comparisons were made using two-tailed paired and unpaired *t*-tests. Mice were assigned to prosocial or selfish groups using one-sample *t*-tests compared to chance (50%). The accepted value for significance was *P* < 0.05. PCA incorporated the following parameters: number of prosocial and selfish responses, decision preference score and latency to respond in the SDM, nose-to-nose contact, body and anogenital sniffing, self-grooming and time spent in the three chamber areas (familiar, unfamiliar and object). Sample sizes and statistical tests are reported in the figure legends. Data distribution was tested using the D’Agostino and Pearson normality test. The experiments reported in this work were repeated independently at least two to four times. For the initial validation of SDM task we replicated the experiment 14 times, which included both naive and virus-injected mice. All exact statistical *P* values can be found in the Source Data files. Individual data points are shown in the figures to reveal the data distribution.

### Reporting summary

Further information on research design is available in the Nature Portfolio Reporting Summary linked to this article.

## Online content

Any methods, additional references, Nature Portfolio reporting summaries, source data, extended data, supplementary information, acknowledgements, peer review information; details of author contributions and competing interests; and statements of data and code availability are available at 10.1038/s41593-026-02292-2.

## Supplementary information

Reporting Summary

## Data Availability

All source data used to generate the figures are available at 10.6084/m9.figshare.31443760 (ref. ^48^).

## Code availability

Custom-written analysis code is available upon request and it will be released in a separate publication.
