# Supplementary material for: Individual differences in prosocial learning are represented in the hippocampal dorsal CA1
Source: Nat Neurosci. 2026 May 12;29(7):1667–79. doi: 10.1038/s41593-026-02292-2 (PMC13337505; doi:10.1038/s41593-026-02292-2)
Supplement: Supplementary file 1 — Reporting Summary [file 41593_2026_2292_MOESM1_ESM.pdf]

nature portfolio

Corresponding author(s): Diego Scheggia

Last updated by author(s): Mar 2, 2026

Reporting Summary

Nature Portfolio wishes to improve the reproducibility of the work that we publish. This form provides structure for consistency and transparency in reporting. For further information on Nature Portfolio policies, see our Editorial Policies and the Editorial Policy Checklist.

Statistics

For all statistical analyses, confirm that the following items are present in the figure legend, table legend, main text, or Methods section.

n/a	Confirmed	
☐	☒	The exact sample size (*n*) for each experimental group/condition, given as a discrete number and unit of measurement
☐	☒	A statement on whether measurements were taken from distinct samples or whether the same sample was measured repeatedly
☐	☒	The statistical test(s) used AND whether they are one- or two-sided<br>*Only common tests should be described solely by name; describe more complex techniques in the Methods section.*
☒	☐	A description of all covariates tested
☐	☒	A description of any assumptions or corrections, such as tests of normality and adjustment for multiple comparisons
☐	☒	A full description of the statistical parameters including central tendency (e.g. means) or other basic estimates (e.g. regression coefficient) AND variation (e.g. standard deviation) or associated estimates of uncertainty (e.g. confidence intervals)
☐	☒	For null hypothesis testing, the test statistic (e.g. *F*, *t*, *r*) with confidence intervals, effect sizes, degrees of freedom and *P* value noted<br>*Give P values as exact values whenever suitable.*
☒	☐	For Bayesian analysis, information on the choice of priors and Markov chain Monte Carlo settings
☒	☐	For hierarchical and complex designs, identification of the appropriate level for tests and full reporting of outcomes
☐	☒	Estimates of effect sizes (e.g. Cohen's *d*, Pearson's *r*), indicating how they were calculated

Our web collection on statistics for biologists contains articles on many of the points above.

Software and code

Policy information about availability of computer code

Data collection	Behavioral data was acquired using Anymaze v. 6.02 (Stoelting) and MED PC-V v. 5.15 (MedAssociates). Fiber photometry data was acquired using signal processor (Doric)
Data analysis	Behavioral data were analyzed using Anymaze v. 6.2 (Stoelting), Boris v.8.6.5 (Unito), DeepLabCut (https://github.com/DeepLabCut/DeepLabCut) and SimBA (https://github.com/sgoldenlab/simba).<br>Fiber photometry data were processed and analyzed using a custom-written MatLab code that will available before publication.<br>c-Fos expression was quantified using Fiji v. 2.5.0.<br>Figures were plotted using GraphPad Prism 9.

For manuscripts utilizing custom algorithms or software that are central to the research but not yet described in published literature, software must be made available to editors and reviewers. We strongly encourage code deposition in a community repository (e.g. GitHub). See the Nature Portfolio guidelines for submitting code & software for further information.

Data

Policy information about availability of data

All manuscripts must include a data availability statement. This statement should provide the following information, where applicable:

- Accession codes, unique identifiers, or web links for publicly available datasets
- A description of any restrictions on data availability
- For clinical datasets or third party data, please ensure that the statement adheres to our policy

All source data used to generate the figures are available at: 10.6084/m9.figshare.31443760

Research involving human participants, their data, or biological material

Policy information about studies with human participants or human data. See also policy information about sex, gender (identity/presentation), and sexual orientation and race, ethnicity and racism.

Reporting on sex and gender	n/a
Reporting on race, ethnicity, or other socially relevant groupings	n/a
Population characteristics	n/a
Recruitment	n/a
Ethics oversight	n/a

Note that full information on the approval of the study protocol must also be provided in the manuscript.

Field-specific reporting

Please select the one below that is the best fit for your research. If you are not sure, read the appropriate sections before making your selection.

☒ Life sciences ☐ Behavioural & social sciences ☐ Ecological, evolutionary & environmental sciences

For a reference copy of the document with all sections, see nature.com/documents/nr-reporting-summary-flat.pdf

Life sciences study design

All studies must disclose on these points even when the disclosure is negative.

Sample size	No statistical methods were used to predetermine sample size. Sample size was determined based on our experience and the sample size used in related research and literature (References: 8, 18).
Data exclusions	Two mouse was excluded from data collection because it showed little motivation to engage in nose poke responses (fewer than 10 total pokes), two were excluded because viral expression patterns were not appropriate (outside the target region) and two were excluded due to fiber misplacement and loss. OBS and DEM were trained in pairs however in few cases we could not quantify data due to technical problems related to c-Fos antibody and immunoistochemistry procedures.
Replication	All the experiments were reproduced in at least two independent experiment in different cohort of animals.
Randomization	Within each cage, mice were randomly selected and assigned to DEM or OBS groups, and to control or hM4D groups, and to control or stGtACR2 or hChR2, in the SDM task.
Blinding	Data collection were not performed blind to the conditions of experiments. Data analyses was performed by experimenters blind to the manipulations.

Reporting for specific materials, systems and methods

We require information from authors about some types of materials, experimental systems and methods used in many studies. Here, indicate whether each material, system or method listed is relevant to your study. If you are not sure if a list item applies to your research, read the appropriate section before selecting a response.

Materials & experimental systems

n/a	Involved in the study
☐	☒ Antibodies
☒	☐ Eukaryotic cell lines
☒	☐ Palaeontology and archaeology
☐	☒ Animals and other organisms
☒	☐ Clinical data
☒	☐ Dual use research of concern
☒	☐ Plants

Methods

n/a	Involved in the study
☒	☐ ChIP-seq
☒	☐ Flow cytometry
☒	☐ MRI-based neuroimaging

Antibodies

Antibodies used

For immunohistochemistry analyses, the following primary antibodies were used: rabbit anti-cFos (#2250, Cell Signaling, dilution 1:500) rabbit anti-DsRed (632496, Takara; dilution: 1:1,000), and rabbit anti-PCP4 (HPA005792; Sigma-Aldrich; dilution 1:200). The following secondary antibodies were used: goat anti-rabbit-Alexa488 (A-11034, Invitrogen; dilution: 1:1,000) and goat anti-mouse Alexa647 (A-21235, Invitrogen; dilution: 1:1,000).

Validation

Quality control information and relevant citations are available at manufacturer's website:
anti-cFos (#2250, Cell Signaling) https://www.cellsignal.com/datasheet.jsp?productId=2250&images=1&size=A4
anti-DsRed (632496, Takara) https://www.takarabio.com/learning-centers/gene-function/fluorescent-proteins/fluorescent-protein-antibody-citations/rfp-antibody-citations);
anti-PCP4 (HPA005792; Sigma-Aldrich) https://www.sigmaaldrich.com/IT/en/product/sigma/hpa005792?srsltid=AfmBOoqJyWwevYso4X7hWdkT_n4nKZEozj3iRkZbfL0JtPpVXKsdO5Wm

Animals and other research organisms

Policy information about studies involving animals; ARRIVE guidelines recommended for reporting animal research, and Sex and Gender in Research

Laboratory animals

C57BL6/J and Fos2A-iCreER;TRAP2 male and female mice, two to five months old. Animals were housed two to four per cage in a climate-controlled facility (temperature 22±2 °C, humidity 45-65%), with ad libitum access to food and water throughout, and with a 12-hour light/dark cycle (7pm/7am schedule).

Wild animals

This study did not involve wild animals.

Reporting on sex

We tested males and females mice in the SDM in 14 independent experiments (Figure 1 and Extended Data Fig.1). For the initial assessment (Extended Data Fig.1) data have been shown separated between males and females. Since we did not detect any difference in observational learning, data from males and females have been pooled in later experiments.

Field-collected samples

The study did not involve samples collected from the field.

Ethics oversight

All procedures were approved by the Italian Ministry of Health (permits n. 191/2020-PR and 200/2022-PR) and local Animal Use Committee and were conducted in accordance with the Guide for the Care and Use of Laboratory Animals of the National Institutes of Health and the European Community Council Directives

Note that full information on the approval of the study protocol must also be provided in the manuscript.

Plants

Seed stocks

Report on the source of all seed stocks or other plant material used. If applicable, state the seed stock centre and catalogue number. If plant specimens were collected from the field, describe the collection location, date and sampling procedures.

Novel plant genotypes

Describe the methods by which all novel plant genotypes were produced. This includes those generated by transgenic approaches, gene editing, chemical/radiation-based mutagenesis and hybridization. For transgenic lines, describe the transformation method, the number of independent lines analyzed and the generation upon which experiments were performed. For gene-edited lines, describe the editor used, the endogenous sequence targeted for editing, the targeting guide RNA sequence (if applicable) and how the editor was applied.

Authentication

Describe any authentication procedures for each seed stock used or novel genotype generated. Describe any experiments used to assess the effect of a mutation and, where applicable, how potential secondary effects (e.g. second site T-DNA insertions, mosiacism, off-target gene editing) were examined.
